# Exploring the Species Diversity of Edible Mushrooms in Yunnan, Southwestern China, by DNA Barcoding

**DOI:** 10.3390/jof7040310

**Published:** 2021-04-17

**Authors:** Ying Zhang, Meizi Mo, Liu Yang, Fei Mi, Yang Cao, Chunli Liu, Xiaozhao Tang, Pengfei Wang, Jianping Xu

**Affiliations:** 1 State Key Laboratory for Conservation and Utilization of Bio-Resources in Yunnan, and Key Laboratory for Southwest Microbial Diversity of the Ministry of Education, Yunnan University, Kunming 650032, China; yzh_1210@hotmail.com (Y.Z.); mmz1761701533@163.com (M.M.); yangliu126377@163.com (L.Y.); mifei99@126.com (F.M.); caoyang8352@163.com (Y.C.); chunli.liu@foxmail.com (C.L.); xiaozhao200809@126.com (X.T.); 15288453604@sina.cn (P.W.); 2 School of Life Science, Yunnan University, Kunming 650032, China; 3 Department of Biology, McMaster University, Hamilton, ON L8S 4K1, Canada

**Keywords:** cryptic species, interspecies genetic divergence, ITS barcoding, poisonous mushrooms, taxonomy

## Abstract

Yunnan Province, China, is famous for its abundant wild edible mushroom diversity and a rich source of the world’s wild mushroom trade markets. However, much remains unknown about the diversity of edible mushrooms, including the number of wild edible mushroom species and their distributions. In this study, we collected and analyzed 3585 mushroom samples from wild mushroom markets in 35 counties across Yunnan Province from 2010 to 2019. Among these samples, we successfully obtained the DNA barcode sequences from 2198 samples. Sequence comparisons revealed that these 2198 samples likely belonged to 159 known species in 56 different genera, 31 families, 11 orders, 2 classes, and 2 phyla. Significantly, 51.13% of these samples had sequence similarities to known species at lower than 97%, likely representing new taxa. Further phylogenetic analyses on several common mushroom groups including 1536 internal transcribed spacer (ITS) sequences suggested the existence of 20 new (cryptic) species in these groups. The extensive new and cryptic species diversity in wild mushroom markets in Yunnan calls for greater attention for the conservation and utilization of these resources. Our results on both the distinct barcode sequences and the distributions of these sequences should facilitate new mushroom species discovery and forensic authentication of high-valued mushrooms and contribute to the scientific inventory for the management of wild mushroom markets.

## 1. Introduction

Fungi make up a remarkably diverse kingdom in terrestrial ecosystems and establish important interactions with plants and animals. Despite recent progress in fungal systematics and taxonomy [1,2,3,4], less than 5% of the estimated 2.2 to 3.8 million species of extant fungi have been described [5]. With the development of metagenome sequencing and other culture-independent methods, an average 7.8–8.8 times of fungal operational taxonomic units (OTUs) have been discovered versus that of culture-dependent methods, resulting in an estimated range of the total fungal diversity to about 12 million (11.7–13.2) species [6]. However, there are some variations in the estimates of total fungal diversity on the earth. For example, a conservative estimate based on ITS2 metabarcoding suggested a total of about 6.28 million fungal species [7]. Regardless, there is broad consensus among fungal biologists that most of the global fungal diversity remains to be described [8]. Over the last three decades, molecular methods have revolutionized our understanding concerning the phylogenetic relationships among fungi and have substantially altered the traditional primarily morphology-based classification system [9,10]. To overcome the difficulties including the scarcity and ambiguity of morphological characteristics in identification, especially for closely related species, DNA sequences have become increasingly popular for species delimitation and identification [11,12]. Indeed, a number of diagnostic tools based on sequence variations have been developed for important groups of fungi [13,14,15].

Edible mushrooms are a large and fascinating group of fungi—according to a recent review, 3283 mushroom species have been confirmed as edible or conditionally edible, accounting for ~20% of all mushroom taxa recorded in the global sources [16]. These edible mushrooms include mycorrhiza formers, plant pathogens, and saprotrophs. They produce a diverse array of fruiting bodies of different sizes, colors, and shapes. Some of the common edible mushrooms include boletes (e.g., *Boletus edulis*), chanterelles (*Cantharellus cibarius*), truffles (*Tuber indicum*), polypores (*Ganoderma lucidum*), matsutake (*Tricholoma matsutake*), coral-like mushrooms (Clavariaceae), sulfur-shelf fungi (*Laetiporus* spp.), caterpillar fungi (*Ophiocordyceps sinensis*), morels (*Morchella* spp.), puffballs (*Lycoperdon* spp. and *Calvatia* spp.), Caesar’s mushroom (*Amanita caesarea* s.l.), and these can often be easily recognized by trained eyes. The edible mushrooms are consumed by humans for their nutritional and medicinal values [17]. Many of them are highly prized and priced even in their endemic regions [18]. Due to its unique climate and geological diversity, southwestern China (especially Yunnan Province) is recognized as one of the world’s 34 biodiversity hotspots, including that for fungal diversity [19]. For example, during the past decade, from 2010 to 2019, more than 1241 new species of large fungi (including lichens) were published using type specimens found in China [20]. Among these, 429 (34.6%) were from Yunnan Province [20]. Indeed, it is estimated that more than 40% of the world’s and 90% of the Chinese edible mushrooms (about 900 species) grow in Yunnan [21,22]. Some of the well-known edible mushroom species in Yunnan Province include *T. matsutake* (nicknamed the “King of Mushrooms” by some), *Tuber indicum* complex (the underground “Black Diamond”), famous Chinese traditional medicinal mushrooms *Poria cocos* and *Ganoderma lucidum*, and the local favorites *Termitomyces clypeatus*, *Russula virescens*, *Boletus edulis* s.l., and *Thelephora ganbajun*, etc. However, in contrast to the abundant wild edible mushroom resources, the efforts to understand Yunnan’s edible mushroom resources remain limited. For example, aside from a few species, little is known about the geographic structuring, cryptic speciation, and even the number of species of mushrooms that are frequently consumed by the local people [21,23].

Aside from helping to understand the basic biodiversity of mushrooms, molecular surveys of wild edible mushrooms can also provide rapid diagnostic tools for these species and guide their effective utilization and conservation [24,25]. Although Yunnan’s great wild edible fungal resources have played an important role in the edible wild mushroom trade market and the local economy, due to the difficulties associated with artificial cultivation of most wild edible mushrooms and the rising prices of some species, it is becoming increasingly evident that the species and genetic resources of many indigenous wild mushrooms are overexploited. In addition, the significant price differences for some expensive species such as matsutakes, chanterelles, boletes from different parts of the world create conditions for counterfeiting—locally and globally. For example, the Japanese favorite matsutake is a loosely defined species complex in the genus *Tricholoma*. The “true matsutake” is *T. matsutake*, with *Tricholoma magnivelare* and several others considered “matsutake allies” that are also consumed in both Japan and elsewhere but are priced differently. Similarly, although morphologically similar to *T. matsutake*, a more distantly related species *Tricholoma bakamatsutake* is not typically consumed and is commonly called the “fool’s matsutake” or “false matsutake” but can also be found in mushroom markets [23]. Therefore, the price differentiation and unregulated labeling in most local mushroom markets create conditions for counterfeiting. Molecular markers will be able to provide reliable signatures for species, geographic population, and even strain authentication [26].

Furthermore, it is estimated that China has about 480 poisonous species of mushrooms; many of these are also found in Yunnan [27]. With increasing consumer demands but a lack of sufficient knowledge to distinguish many mushrooms, many locals frequently face the risk of eating poisonous mushrooms that are morphologically similar to edible ones. As reported from China Centre of Disease Control and Prevention and China National Center for Food Safety Risk Assessment, there were 7331 mushroom poisoning cases, causing 708 death during 2010–2019 in China [28]. Yunnan was ranked first in both the number of people poisoned by wild mushrooms and in the number of deaths from mushroom poisoning from January to August in 2019. For instance, eating a previously undescribed species of mushroom, which was later named *Trogia venenata,* has been implicated as a risk factor for the Yunnan sudden unexplained death (SUD) [29]. This mushroom is morphologically similar to an edible mushroom called “Badanchai” by the locals, with “Badanchai” including several different species such as *Schizophyllum commune* and *Pleurotus* sp. At certain developmental stages, these mushrooms cannot be easily distinguished from each other by untrained eyes [30].

Accurate species delimitation and documentation are vital for assessing species diversity of mushrooms and helping with sustainable utilization and management of genetic resources. However, the speciose nature of many groups of wild edible mushrooms makes the interpretation of their morphological features a perpetual challenge for many mycologists. Thus, having a reliable DNA sequence-based identification for these mushrooms is essential for their correct identification. Indeed, molecular studies have recently shown the existence of many ‘‘cryptic’’ fungal species, which cannot be distinguished morphologically [13,31,32,33], stressing that molecular information is indispensable for the identification and description of these species and indeed fungal species in general. The internal transcribed spacers (ITSs) of nuclear ribosomal RNA repeat units have frequently been used to identify fungal species. The multi-copy nature, obvious gaps in sequence identity between many closely related species, and the availability of conserved primers flanking the ITS regions makes ITSs one of the most frequently used barcode markers in the field [34,35,36,37,38,39,40,41]. Up to now, however, the large-scale ITS sequence approach has not been used widely for identifying edible mushrooms, except in a few species and their allies [42], e.g., truffles [43], morels [44], matsutakes [45,46], oyster mushrooms [47], boletes [48], and lethal *Amanita* spp. [49,50]. There have been several sporadic mushroom molecular diversity surveys of local grocery stores in New York [51], London [52], and mushroom dietary supplement from a company called New Chapter, Inc. (Brattleboro, VT, USA) [53]. However, most of these surveys are geographically limited and had relatively small sample sizes. Thus far, most surveys of edible mushroom species in Yunnan markets have been mainly based on morphological features and most scientific names used for describing the edible mushrooms in Yunnan have been borrowed from those of similar species in Europe and North America. However, the situation has gradually changed. Indeed, for several groups of edible mushrooms with significant economic and ecological importance, DNA sequences have helped in revealing extensive diversity and endemism of mushrooms in Yunnan and providing evolutionary connections with species from other regions of the world, including for species in *Boletus* [54,55], *Pleurotus ostreatus* species complex [56], *Flammulina* [57], *Hydnum* [58], *Armillaria* [59], and *Amanitaceae* [60]. To help uncover the true edible mushroom diversity in Yunnan, we surveyed the diversity of wild edible mushrooms in Yunnan Province. Specifically, we aim to address the following two questions: (1) How many known and novel (cryptic) species of mushrooms are usually found in the local markets from Yunnan? and (2) are ITS sequences useful markers for edible mushroom species identification in Yunnan markets?

## 2. Materials and Methods

### 2.1. Sampling

**Figure 1:**
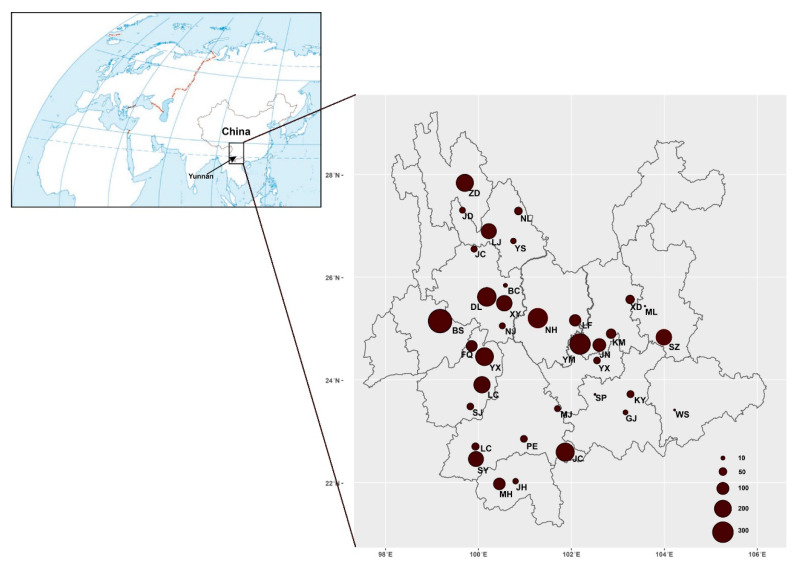
Geographic distribution of all the mushroom samples collected in markets in Yunnan, southwestern China. The sizes of pie charts are proportional to the sample sizes. The full names of each sampling site were indicated in Table S1.

Mushroom samples were collected in 35 counties distributed across Yunnan Province (Table S1, Figure 1), southwestern China. These samples were acquired from local farmers’ markets, specialized mushroom markets, mushroom hunters in forests, and roadside mushroom sellers. The obtained mushroom samples represent almost all the common mushrooms sold and eaten by local people during 2010–2019. In total, 1–30 individuals from each species from each of the sites were collected. For a few commonly found mushrooms, we obtained relatively large sample sizes from each geographical population whenever possible to help identify potential geographically specific genetic elements of the species within Yunnan. All of the mushroom fruiting bodies were dried at 60 °C in a fruit drier overnight and a 1 cm^3^ section was cut and stored in an air-tight Ziploc bag containing silica gel for DNA extraction and subsequent DNA sequence analyses. The dried fruiting bodies were deposited in the Laboratory for Conservation and Utilization of Bio-resources of Yunnan University under number (YMF5.00001-5.03585); the first 2198 specimens are those associated with clean ITS sequences.

### 2.2. DNA Extraction, PCR Amplification, and Sequencing

Genomic DNA was extracted from fruiting bodies using the Cetyltrimethylammoniumbromide (CTAB) method with minor modifications [61]. The extracted individual genomic DNA of each specimen was preserved in deionized water and frozen at −20 °C until used. All samples in this study were analyzed for their sequences at the internal transcribed spacer (ITS) regions. Primer pairs ITS5 (5′GGAAGTAAAAGTCGTAACAAGG3′) and ITS4 (5′TCCTCCGCTTATTGATATGC3′) were used to amplify the complete ITS [41]. The parameters for PCR amplifications are as follows: 1 min initial denaturation at 95 °C, followed by 30 cycles of 1 min denaturation at 94 °C, 1 min primer annealing at 50 °C, 1.5 min extension at 72 °C, and a final extension period of 10 min at 72 °C. The purified PCR products were sequenced bidirectionally by BGI Co. Ltd. (Shenzhen, China) with the same primers that were used for amplification.

### 2.3. Data Analysis

#### 2.3.1. Species Assignment

For each mushroom specimen, its complete ITS sequence was assembled using sequences of both strands through Seqman (DNAStar package). During sequence assembly, sequence chromatograms were manually checked, which allowed the identification of heterozygous sites. All sequences were aligned using ClustalX 1.83 [62] and manual adjustments were made to improve the alignments by the BioEdit 7.0.9 software [63]. Since around 30% of the fungal ITS sequences in GenBank have been found to be problematic [64], to verify the taxa identities of the obtained ITS sequences, a Basic Local Alignment Search Tool (BLAST) search against the sequences deposited on curated UNITE database (http://unite.ut.ee/, accessed on 31 March 2021) was performed on the aligned sequences. Species discrimination was considered successful if the ITS sequence of an individual had a top matching hit of only a conspecific individual with binominal names. Poor matches over short overlapping sequences (<80% coverage) among the query and database sequences were excluded from subsequent taxa identity analyses.

In our BLAST searches against both the GenBank and UNITE databases, all our sequences were only compared with ITS sequences associated with binomial species names, while those from metabarcoding studies without individual fungal specimen identifications were excluded. The identified taxa genus and species names were compared with those in the latest published outline of fungi and fungus-like taxa [10]. The strains whose ITS sequences had the highest sequence identities below 97% to a known species were analyzed using further phylogenetic comparisons.

#### 2.3.2. DNA Barcoding Assessment

Three criteria were used to assess the potential of ITS as a DNA barcode of edible mushrooms in markets in Yunnan Province, southwestern China—(1) the universality of primers for PCR amplification, (2) the quality of DNA sequence, and (3) the barcode gap. The universality of PCR was assessed simply based on the success rates of PCR amplification. The quality of DNA sequence refers to the readability of the sequencing chromatographs to generate clean sequences. For samples with failed PCR amplification or with obtained low-quality DNA sequences, DNA extraction, PCR amplification, and/or DNA sequencing were repeated up to three times. The barcode gap was evaluated by the frequency distribution of the intra- and interspecific distances [65] with Excel (Microsoft, Seattle, WA, USA). Genetic distances were calculated using the Kimura 2-parameter (K2P) model [66] in MEGA 6.06 [67]. The analyses were performed as follows. First, for each dataset, the mean and maximum intraspecific distances and interspecific distances were calculated by comparing the minimum distance between each species and its sister taxon in the same genus. Second, pairwise distances were similarly calculated at the species, genus, family levels, respectively, based on our own sequences, plus the reference ITS sequences from known species.

#### 2.3.3. Phylogenetic Species Identification in Selected Taxa

To detect whether the divergent lineages revealed by the ITS sequences represented potentially phylogenetic species (cryptic species) in genera *Boletus* and allies, *Cantharellus cibarius* species complex, *Lactarius, Lyophyllum, Russula virescens* ally, *Termitomyces clypeatus* species complex, and *Thelephora ganbajun*, our ITS sequences were used as queries to retrieve closely related sequences (>90% sequence identities) with comparable lengths from GenBank and the UNITE database. Only related sequences associated with binominal species names supported by morphological and molecular evidence were selected as references for our comparisons (Table S2).

First, all ITS sequences obtained from our specimens and those from GenBank representing the diversity of species within each of the aforementioned fungal groups were aligned by using MAFFT 6.0 [68] and checked manually by BioEdit 7.0.9 [63]. Ambiguous positions at the two ends of each gene fragment were excluded from the analyses. Maximum likelihood (ML) and Bayesian inference (BI) analyses were conducted by using RAxML 7.2.6 [69] and MrBayes 3.1.2 [70], respectively. ML analyses were run with all parameters set to the default settings and the bootstrap analysis was run with 1000 replicates. BI analysis consisting of four simultaneous Markov chain Monte Carlo (MCMC) chains (three heated chains and a cool chain) was run by setting generations to 10 million with the value of stop set to 0.01. Trees were sampled every 1000 generations. Finally, the initially sampled trees were discarded, and the remaining trees were used to calculate Bayesian posterior probabilities (BPP) in a 50% majority-rule consensus tree. Provisional species were considered discriminated if all individuals of a species formed a monophyletic group [71].

Second, intraspecific variations of ITS sequences among each of several known species were compared to define a threshold value of species limits. Meanwhile, each terminal branch of ITS phylogenetic trees was treated provisionally as one species and used to calculate putative inter- and intraspecific variation with the known species. Then, the provisional species corresponding to each divergent lineage with high statistical support and with a sequence divergence from existing species at greater than the threshold value between known sister species in the genus were accepted as a valid phylogenetic species. Alternatively, if the divergence value was lower than the threshold value between existing sister species, the clades would be considered as belonging to the same species [48]. Similarly, within our samples, if there were multiple lineages within a monophyletic group that showed sequence divergence greater than the divergence value of known sister species in the same genera/family, they would be treated as belonging to two or more new phylogenetic species.

## 3. Results

### 3.1. Sequencing

A total of 3585 mushroom samples were collected from 35 counties across Yunnan Province from 2010 to 2019 (Figure 1). At each site, at least one sample was obtained for each morphologically distinct mushroom. For the common mushrooms, multiple specimens of each morphological species were obtained at all sites. Among these 3585 samples, genomic DNA was successfully obtained from 3381 samples, yielding a 94.3% success rate.

The ITS gene fragment was amplified from all of the 3381 samples. Some of these samples required more than one try to obtain PCR product, often through using different PCR amplification conditions. The success rates of PCR amplification and the frequency of samples showing high-level heterozygosity and causing unreadable sequences for the commonly appeared species (those with sample size over 20) are presented in Table 1. Overall, samples in the genera *Ramaria*, *Termitomyces*, and *Cantharellus* had low ITS amplification success rates and high frequencies of heterozygosity within their ITS sequences. For example, *Termitomyces* has the lowest success rates of PCR amplification in samples from Ganlanwan (16.67%). Long tracks of heterozygotic sites were very frequently observed in three genera—*Ramaria*, *Termitomyces*, and *Cantharellus*. For example, two species complexes, the *Cantharellus cibarius* species complex and the *Ramaria botrytis* species complex, had 45.95% and 18.92% ITS sequences, having large numbers of heterozygotic sites starting from either ITS1 or ITS2 regions or both and with clean sequences ranging only from 76–328 bp for individual specimens. By sequencing from both directions, for some of these samples, we were able to obtain longer stretches of sequences. In the *Termitomyces clypeatus* species complex, 7.4% and 1.2% ITS sequence chromatographs had long tracks of heterozygotic sites starting from ITS1 and ITS2 regions, respectively, and we were only able to obtain clean sequences of 70–148 bp for these samples. While sufficient for certain analyses to identify the specimen on the higher taxonomic levels, these very short sequences are often insufficient for species identification or for phylogenetic studies. Excluding the 1183 specimens that failed to obtain high-quality ITS sequences (~35% of the 3381 genomic DNA preps), our final dataset included 2198 full ITS sequences for downstream analyses, including both BLAST searches against both the GenBank and the UNITE databases and phylogenetic analyses with their closely related known species.

### 3.2. Molecular Species Identification

#### 3.2.1. Species Estimation

Based on morphological characteristics and experiences of local people, all specimens were roughly identified to 41 species. However, according to ITS sequences data, the species number is far more than that.

At present, a consensus fungal-wide cutoff value to demarcate intra- from interspecific ITS sequence variability has not been determined yet [40]. Here, we first applied the commonly used threshold of >97% sequence identity as within-species variation as the first approximation in our analyses. From the total 2198 samples from which we successfully obtained their ITS sequences, we found a total of 58 species that matched existing species (representing 48.86% of the total ITS sequences) when the within-species ITS sequence similarity was set at >97%. For samples that failed to classify to species level based on 97% sequence identity, we progressively used other threshold values 95–97%, 90–95%, and lower than 90% to detect additional species. The reduced threshold values allowed us to identify an additional 17 (13.33% of the ITS sequences), 30 (22.38% of the ITS sequences), and 54 (15.42% of the ITS sequences) known species that, respectively, matched our samples. Thus, using the very loose criteria, we found at least 159 species with binomial names matching our samples (Table 2). Together, these 159 species belonged to 56 different genera, 31 families, 11 orders, 2 classes, and 2 phyla (Table 2). Based on a newly published checklist of Chinese macrofungal resources [27] and the new evidence-based classification system on the world’s edible mushroom species [16], we found that most of the species (85/159) are known edible fungi. However, nine of them have been reported as poisonous, and nine of them are edible but mainly used for medicinal purposes. These medicinal mushrooms play important roles in treating cancer, eczema, inflammation, etc. The remaining 55 species are unconfirmed on their edibility, and 20 of them are new records to China.

Representative ITS sequences for all the OTUs in Table 2 have been submitted to GenBank with the following accession numbers KU165834-KU165846, MW874484-874578, MW893254-MW893269, MW930735, and MW932679-MW932711. The sample code, GenBank accession numbers, and their corresponding OTU identifications in the UNITE and GenBank are presented Supplementary Table S4.

#### 3.2.2. Potential New Species Based on Adjusted Criteria

In the estimates presented above, we used hard cutoff values based on full-length ITS sequence identity (at >97%; 95–97%; 90–95%; and <90%) to determine the putative number of species sold in the edible mushrooms market. However, we recognize that this approach cannot be universally applied to all fungal groups. In fungi, well-known sister species in some groups have very similar (>99% sequence identity) or even identical ITS sequences. In this section, we use an alternative approach to estimate the potential number of species in several fungal groups in which either (1) both our sample sizes are large and abundant taxonomic information and ITS sequence data are already available for the specific genera, or (2) species closely related to our sampled market mushrooms in Yunnan are found. The large sample sizes allow us to obtain both intra- and interspecies ITS sequence divergence. Specifically, we focused on analyzing our samples from the following genera/groups of fungi: *Boletus* and allies, *Cantharellus cibarius* species complex, *Lactarius*, *Lyophyllum*, *Russula virescens* allies, *Termitomyces clypeatus* species complex, and the *Thelephora ganbajun* species complex.

In these analyses, because of the large sample sizes for each group of mushrooms, we first constructed a phylogenetic tree for each group, followed by identifying and removing the duplicated sequences from each geographic region (county) in each dataset. In each finalized dataset, only one representative strain was kept for each unique ITS sequence from each county. Reference sequences were similarly treated. The adjusted reference sequences were then used to estimate both the intraspecific ITS sequence variation and pairwise interspecific ITS sequence variations for all the known species in each group. These estimates help to determine the critical values for our subsequent comparisons. To prepare for the comparisons, we similarly calculated the ITS sequence divergence between our sequences with each other and with those of the known species. The summary results of our comparisons are shown in Table 3. Specifically, the number of provisional species based on this method for these seven groups of mushrooms (110 distinct monophyletic branches of our strains) is about 46% more than those of ITS Blast described above (59) (Figures S1–S7). When the highest intraspecific ITS sequence variation within each of the above seven groups (i.e., the most conservative approach) was adopted as the cutoff value for new species identification (Table 3 and Table S3), up to six new species were identified for each taxonomic unit, with a total 20 additional provisional species in the selected 1536 ITS sequences.

Here, we use the *Cantharellus cibarius* species complex (CCSC) as an example to further illustrate the potential diversity of mushroom species in Yunnan markets. In this species complex, there are six recognized species in our samples. The intraspecific variations of ITS sequences within each of the six known species ranged from 0 to 0.028. Thus, we used 0.028 as the presumptive cutoff value for the phylogenetic species identification in CCSC using ITS sequences. Our samples of *Cantharellus* sp. 2–6 have variations lower than or equal to this cutoff with their closely related known species (0.016, 0.008, 0.028, 0.016, and 0.016, respectively) (Figure S2 and Table S3), so they may belong to their closely related species. However, *Cantharellus* sp. 1 showed divergences higher than 0.028 (0.034) with their closely related known species *Cantharellus lateritius*.

Since many *Cantharellus* specimens have highly variable ITS sequences within individual strains, the ITS region has been considered not suitable as the barcode for this genus. Instead, the translation elongation factor 1-alpha (TEF-1) has been adopted as the alternative DNA barcode for species delimitation in this genus [72,73]. The intraspecific variations of TEF-1 sequences within each of the 22 known species in this species complex ranged from 0.000 to 0.008. To clarify the potential number of species within CCSC in our sample further, we obtained the TEF-1 sequences from all our samples in CCSC. The phylogenetic analyses of TEF-1 sequences representing the five putative novel species identified based on ITS sequences are presented in Figure S8. Our analyses showed a range of variations between our specimens and those of known species, with sequence divergence between our putative novel species and their closest known species being similar to or greater than most of the known sister species within CCSC (Figure S8). However, if we use the largest intraspecific TEF-1 sequence variation (0.008) as the presumptive cutoff value for the phylogenetic species identification in CCSC using TEF-1 sequences, four of the five putative species would be considered as synonyms with existing species, and only *Cantharellus* sp.5 in Figure S8 would be considered as a novel species. Therefore, using the largest intraspecific sequence variation as the cutoff, both the ITS and TEF-1 sequence results suggest that there is at least one cryptic species within the CCSC in mushroom markets in Yunnan [74]. It is worth mentioning that using the largest intraspecific sequence variation as the cutoff for new species identification in a genus and species complex is a very conservative approach. For example, if we were to 0.008 as intraspecies variation cutoff for CCSC, the current 22 recognized species would be reduced to 12 species.

### 3.3. Intraspecific Variation, Interspecific Variation, and DNA Barcoding Gaps

**Figure 2:**
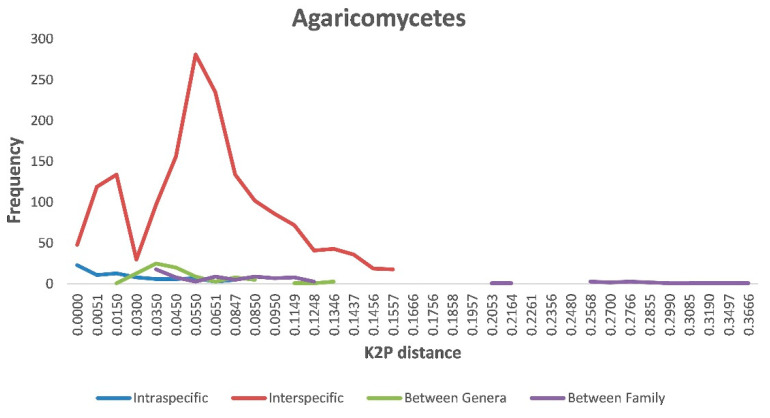
Relative distribution of genetic divergence at intra- and interspecies, between genera, and between family levels in Agaricomycetes.

Our BLASTn searches identified a total of at least 159 species that belonged to 56 genera, 31 families, 11 orders, 2 classes, and 2 phyla. Using this dataset that included ITS sequences from both our specimens and the reference sequences of their closely related known species, we further investigated sequence variations at various levels within Agaricomycetes. Specifically, we inferred the range of genetic distances within individual species, between species within a genus, between genera within a family, and between families. Our comparisons showed that the greatest intraspecific K2P distance within Agaricomycetes was 0.104. In comparison, interspecific divergence within individual genera ranged from 0 to 0.16 within Agaricomycetes, with most distances around 0.055. The intra- and interspecies genetic divergence greatly overlapped each other (Figure 2). Similarly, but to different extents, all four K2P distances (intraspecific, interspecific within a genus, intergenera within a family, and interfamily) overlapped with each other within Agaricomycetes (Figure 2).

## 4. Discussion

### 4.1. Species Number Estimation

In this study, with the help of DNA sequence information at the ITS regions, we found about four times of the mushroom species number as that was recognized by locals in markets across Yunnan Province in southwestern China. The finding that about 48.86% of samples could be matched to existing species by above 97% ITS sequence similarity is comparable to ITS barcoding on grocery sold mushrooms, where 50% of the samples were unambiguously assigned Latin binomial names [53]. Our results indicate extensive diversity and reveal evidence of cryptic speciation within many of these mushroom species and species complexes. Among these species, nearly one-third (55) of the species have never been reported on their edibility, and 20 of them are newly recorded in China. Some of them have been listed in the “Red List of China’s Biodiversity—Macrofungi” [75]. The high diversity was supported by another ITS barcoding analysis in which 3 out of 15 pieces of dried Chinese porcini mushrooms in a single commercial packet in London, England represented new species [52], one of which was also later identified in a US grocery store [53]. Furthermore, 9 out of 159 species are known as poisonous mushrooms that can cause severe adverse health effects in people, including deaths (Table 2). These nine species are morphologically similar to edible ones in the families *Amanitaceae, Boletaceae,* and *Gomphaceae*, and with very similar macromorphological characters to *Amanita javanica*, *Boletus edulis*, and *Ramaria botrytis* [27,76]. Together, our analyses indicated a very high diversity of species in local wild mushroom markets, including both endangered and poisonous mushrooms.

For several reasons, species identification in fungi can be very challenging. For edible mushrooms, their fruiting and morphological features of fruiting bodies are influenced by many biotic and/or abiotic factors. It is often difficult to distinguish related species based on morphological features alone, even for experts working on those species. The expanding number of cryptic species as revealed by increasingly more discriminatory molecular markers also complicates species identification in the ever-evolving taxonomic framework [13]. In Yunnan Province, as in many other developing regions and countries, while significant advances in mushroom taxonomy and species diversity have been made, our understanding is still limited and often fragmented. This contrasts with the relatively complete studies of mushroom taxonomy in Europe and North America. For example, the taxonomy of most mushroom species in Yunnan has been mainly based on morphological features and predominantly used names of samples from Europe and North America [48,77]. However, over the past two decades, the use of molecular phylogenetic techniques have greatly contributed and improved studies such as species diversity, systematics, speciation, and migration of higher fungi in Yunnan, including diverse fungal groups, such as those in orders of Agaricales [24,60,78], Boletales [79], Pezizales [80], Polyporales [81], and Russulales [82,83]. Our study builds on those early studies. Using sequences at the DNA barcode ITS and based on estimates of infraspecific ITS sequence variability, we revealed extensive species diversity in Yunnan mushroom markets, including potentially many cryptic and entirely new species [40]. Indeed, the identified sequence divergence between our strains and those of the previously published suggest the potential existence of novel taxonomic groups above species level among these market mushrooms. However, caution should be taken before the final number of novel species from our market surveys is known. For example, some of these divergent ITS sequences may represent previously described species, but no ITS sequence was available. To investigate this possibility formally and name these putative new taxa, obtaining samples from their native ecological niches and comparing them with closely related species on DNA sequences at other genes as well as on both macro- and micromorphological features are needed. Our results here provide the foundations from which to identify where some of those novel taxa may be resided from both geographic and evolutionary perspectives.

Our study here also suggested that we need an improved understanding of the biodiversity of these wild edible mushrooms in order to develop effective conservation and utilization strategies of these resources. For example, population genetic analyses of mushroom samples can help us understand how these mushrooms reproduce in nature and the extent of gene flow among local and regional geographic populations. Sexual recombination is prevalent in natural populations of these mushrooms, and thus, sexual spores of these mushrooms should be allowed to mature and spread to ensure their continued reproductive success in nature [24,25,83]. To help achieve this goal, instead of harvesting all the fruiting bodies, a large number of fruit bodies must be allowed to mature and sporulate to generate mature sexual spores. In addition, since most mushrooms require the accumulation of considerable vegetative mycelia before they can fruit [84], care must be taken during mushroom picking to minimize the disturbance of the vegetative mycelia in situ (mostly underground). For ant nests associated *Termitomyces*, maintaining healthy ant colonies are also essential for their fruiting.

On the other hand, the identification of about a dozen poisonous mushroom species in those markets is extremely concerning. There are several reasons for the presence of poisonous mushrooms in those markets. The first is a lack of market entry standards for wild mushrooms. In most areas in Yunnan, almost all wild-picked mushrooms could end up in the markets. While some mushroom pickers, traders, and sellers are able to identify the common edible mushrooms, most cannot identify all wild mushrooms. Often, the ultimate responsibility for identification falls on individual consumers to decide which ones they are willing to purchase and consume. However, most consumers do not have the required knowledge to distinguish most of the wild-picked mushrooms either. Consequently, mushroom poisoning happens every year in Yunnan. In several northern regions in China, mushroom poisoning has led the local governments to introduce laws that prohibit selling wild mushrooms [85]. Unfortunately, these laws have decimated the local wild mushroom industry and negatively impacted other related businesses. We recognize that completely banning this industry is neither desirable nor even feasible; instead, having broader education and more stringent regulations at multiple levels could help reduce/eliminate mushroom poisoning. The education and regulations will need to be implemented for the pickers, traders, salespeople, and consumers. Our study identified a number of poisonous mushrooms in the markets. In the education campaign, these mushrooms should be highlighted, and all stakeholders should be taught to avoid picking/selling/consuming those mushrooms and their close relatives. In the future, handhold devices may be developed that can directly allow mushroom pickers/traders/salespeople/consumers to identify suspicious mushrooms based on DNA sequences. Our study provides a large number of DNA barcode sequences from which such a system can be developed. In addition, this study identified the methods that worked for most edible mushrooms in specimen collection, DNA extraction, PCR amplification and sequencing of the DNA barcode, editing and submission for DNA barcoding files, and procedures and methods for the analysis of DNA barcoding data in the Yunnan wild mushroom markets.

### 4.2. Feasibility of ITS Sequence as Identification Marker of Wild Mushroom Species

The number of fungal ITS sequence accessions in GenBank was 1,403,564 by 31 March 2021. These ITS sequences covered more than 2500 genera, 15,500 species, and with binomial species, names are associated with ~57% of those sequences [86]. Although still incomplete, and the nomenclature of GenBank accessions not updated when taxonomy is changed, the large database of ITS sequences offers a powerful reference for the initial identification of most fungi. Our study affirms the usefulness of the ITS DNA barcode and the GenBank and UNITE databases for the initial identifications for all of our specimens. These initial identifications may not be precise at the species level, but they can provide a framework at higher levels such as order, class, family, and genera for further explorations. With increasing contributions from fungal biologists and an expanding ITS dataset in GenBank and UNITE, the probability of correct and precise identification for most mushrooms at the species level will continue to increase.

The distance between intraspecific and interspecific sequence variation (the DNA barcoding gap) is among the most important criteria in DNA barcoding practice. When the maximum intraspecific sequence distance is less than the minimum interspecific sequence distance among closely related taxa, these taxa are known to have clear barcode gaps. A clear barcode gap is highly desirable for sequence-based species identification and makes sequence-based species discrimination relatively straightforward. However, we found that the intra- and interspecific genetic divergence greatly overlapped for many known groups of fungi (Figure 2). The overlapping intra- and interspecific levels of sequence divergences likely reflect a number of issues, including (1) different mutation rates at the ITS locus among fungi, (2) different criteria used to define fungal species, and (3) errors in GenBank sequence entries, with wrong/outdated taxonomic affiliations to specific sequences [40]. Indeed, these issues have been known for a long time. Mushroom fungi cover a large number of taxonomic groups, with some sister species showing low ITS sequence divergence, while others showing significant divergence. Indeed, these variations have prevented the broad adoption of a single cutoff value to define inter- vs. intraspecific variations for any locus. Similar to our analyses here (Figure 2), a meta-analysis using publicly available fungal ITS sequences revealed that the weighted percentage of within species’ divergence of all fungi was 2.51%, while that for within Basidiomycotina was 3.33%, both of which show significant overlap with interspecific divergence [40]. In our study, we have used a range of sequence divergence thresholds to estimate species numbers, with some of them far exceeding the typical amount. Consequently, we believe that our estimate of 159 species is an underestimate of the true species number in our collection.

Despite the above-mentioned successes of using ITS sequences to identify the putative diversity of mushrooms from markets, our investigation also revealed a big issue with relying on ITS sequences alone as a barcode for these mushrooms. Specifically, we were unable to obtain clean and/or sufficient ITS sequences from a large number of mushroom specimens. While some of them were due to failed DNA extraction and failed PCR amplification, others in several taxonomic groups had high levels of heterozygosity, making most of the sequence chromatographs unreadable. A number of reasons could have contributed to the unreadable sequences. The nuclear ribosomal RNA gene cluster exists as a variable number of repeats in each genome, and consequently, the ITS regions (ITS1 and ITS2) also exist in many copies in each cell. Through evolution, these copies accumulate different mutations, and if there were insertion or deletion mutations for some copies, the DNA sequence chromatographs based on the genomic DNA could become unreadable. Another reason is hybridization, with the hybrids containing ITS sequences from two divergent ancestors that differ in their ITS sequences. Our analyses here revealed high rates of heterozygotic sites in several groups of fungi, including the *Cantharellus cibarius* species complex, and several species in genera *Termitomyces* and *Ramaria*. In addition, as shown in several groups of mushroom fungi, ITS sequences are not appropriate or sufficient for the identification of species in those groups [54,72,74]. For these fungi, sequences from alternative markers are needed in order to provide precise identifications. Even with alternative DNA barcodes such as TEF-1 for the genus *Cantharellus*, a single-gene-based taxonomy can be problematic. In the case of CCSC, there were notable overlaps between intraspecific and interspecific TEF-1 and ITS sequence variations within and among the known species. This was more broadly observed among basidiomycete fungi (Figure 2). Overall, we believe that while ITS sequencing will likely remain powerful for preliminary identification of most mushroom fungi in the near future, a robust, DNA sequence-based identification will require more complete taxonomic knowledge specific to each group of fungi, a large number of conspecific specimens from as many populations and geographical regions as possible, and DNA sequence information from other gene loci (secondary and supplementary barcoding markers) or whole genomes [40]. Population genetic analyses based on large sample sizes and multiple marker genes (including whole genome sequences) are needed in order to determine whether closely related (cryptic) species are reproductively isolated in nature.

## 5. Conclusions

In summary, this study provides significant new information on mushroom species diversity in the markets in Yunnan, China. Our analyses uncovered extensive diversity, identified several new records, and revealed a large number of potential new species. The data presented here can serve as a foundation for detailed taxonomic studies of many mushrooms from these markets in the future. Our results showed the power of ITS DNA barcoding for accurate species identification of many mushrooms, including the methods that can be used by both specialists and government agencies responsible for monitoring the wild mushroom markets. However, we have also identified the shortcomings of this approach. To make our dataset widely useful, we need to provide detailed descriptions for each of the market mushrooms and develop a web-based service using curated and centralized sequences of ITS and other reference loci. This type of platform could serve as a tool both for market management and for developing policies for the conservation of genetic resources of wild edible mushrooms.

## Figures and Tables

**Table 1:** The success rates of PCR amplification and occurrence of heterozygotic sites of internal transcribed spacer (ITS) fragment in genera with sample size over 20.

Genus	Sample Size	ITS Amplicons	Amplification Success (%)	Heterozygotic Sequences
*Amanita*	26	26	100	0
*Auricularia*	37	33	88.6	0
*Boletus*	94	94	100	0
*Butyriboletus*	27	27	100	0
*Cantharellus*	380	277	72.8	177
*Catathelasma*	22	22	100	0
*Cortinarius*	20	20	100	0
*Hygrophorus*	39	39	100	0
*Lactarius*	102	89	87.2	0
*Leucopaxillus*	26	23	86.2	0
*Lyophyllum*	176	125	71	0
*Ramaria*	252	118	47.2	82
*Russula*	294	281	95.7	0
*Termitomyces*	608	396	65.1	118
*Thelephora*	494	494	100	0
*Tricholoma*	35	35	100	0

**Table 2:** Species identification based on ITS sequence’s best BLAST matches.

Class	Order	Family	Genus	Species	≥97%	95–97%	90–95%	≤90%	Sample Size	Edibility Status	Intraspecific Distance
*Sordariomycetes*	*Hypocreales*	*Cordycipitaceae*	*Cordyceps*	*C. militaris*	4				4	E	0
*Agaricomycetes*	*Agaricales*	*Agaricaceae*	*Agaricus*	*A. rubescens*	2			1	3	E	0.029
				*A. virgineoides*				2	2	P	n/a
		*Amanitaceae*	*Amanita*	*A. caojizong*		1	2	2	5	E	0.099
				*A. citrinoannulata*	1				1	U	n/a
				*A. cupreobrunneus*	3	1			4	E	0.005
				*A. depauperatus*	1				1	U, N	n/a
				*A. imazekii*				6	6	E	0.057
				*A. masasiensis*				5	5	U, N	0.049
				*A. pantherina*				2	2	N	n/a
				*A. pseudoporphyria*			1	1	2	E	n/a
		*Biannulariaceae*	*Catathelasma*	*C. ventricosum*		6	14		20	E	0.012
		*Cortinariaceae*	*Cortinarius*	*C. balteatoalbus*		8	2		10	U	0.077
				*C. caperatus*		3			3	E	0
				*C. flavescentipes*	1		5		6	U, N	0.003
				*C. vernus*				1	1	U	n/a
		*Hydnangiaceae*	*Laccaria*	*L. laccata*	1				1	E	n/a
			*Laccaria*	*L. vinaceoavellanea*		8	4	1	13	E	0.029
		*Hygrophoraceae*	*Hygrophorus*	*H. agathosmus*			3		3	E	0.002
				*H. hypothejus*				1	1	E	n/a
				*H. purpurascens*		13	1		14	E	0.002
				*H. russula*			1		1	E	n/a
		*Hymenogastraceae*	*Naucoria*	*N. fellea*				1	1	U, N	n/a
			*Psilocybe*	*P. semilanceata*				14	14	P	0.061
		*Lyophyllaceae*	*Lyophyllum*	*L. decastes*			1	1	2	E	n/a
				*L. fumosum*	5	5	6		16	E	0.084
				*L. shimeji*	77	4		3	84	E	0.048
			*Termitomyces*	*T. bulborhizus*			10		10	E	0.003
				*T. clypeatus*	13		176		189	E	0.011
				*T. eurrhizus*	66				66	E	0.003
				*T. heimii*	5	4			9	E	0.012
				*T. medius*			5	8	13	E	0.008
				*T. microcarpus*	1		44	89	134	E	0.055
				*T. radicatus*			4		4	E	0.045
			*Tephrocybe*	*T. ancida*		3			3	U, N	0.04
		*Omphalotaceae*	*Lentinula*	*L. lateritia*		1		2	3	U, N	0.065
		*Physalacriaceae*	*Hymenopellis*	*H. radicata*				1	1	E	n/a
		*Pleurotaceae*	*Pleurotus*	*P. giganteus*	3				3	U	0.001
		*Pseudoclitocybaceae*	*Pseudoclitocybe*	*P. cyathiformis*				1	1	E	n/a
		*Tricholomataceae*	*Tricholoma*	*T. albobrunneum*	3	1			4	M	0.009
				*T. dulciolens*				2	2	U, N	n/a
				*T. equestre*		1	1		2	M	n/a
				*T. joachimii*	3		1		4	U	0.014
				*T. matsutake*	18				18	E	0.002
				*T. stans*	2	1			3	E	0.001
				*T. terreum*	4				4	E	0.006
	*Agaricales genera incertae sedis*		*Clitocybe*	*C. nebularis*	24		1	1	26	E	0.101
			*Lepista*	*L. sordida*	2				2	E	n/a
			*Leucocybe*	*L. connata*	2		1		3	M	0.013
	*Auriculariale*	*Auriculariaceae*	*Auricularia*	*A. cornea*		22	8	1	31	E	0.028
	*Boletales*	*Boletaceae*	*Aureoboletus*	*A. moravicus*				1	1	U	n/a
			*Austroboletus*	*A. gracilis*				1	1	U, N	n/a
			*Baorangia*	*B. bicolor*		3	2		5	U	0.02
			*Boletus*	*B. aereus*			1		1	E	n/a
				*B. eastwoodiae*				2	2	U	n/a
				*B. edulis s.l.*				2	2	E	n/a
				*B. pseudosulphureus*				4	4	E	0.022
				*B. reticulatus*	1	15	45	6	67	E	0.075
				*B. rhodopurpureus*				1	1	U	n/a
				*B. satanas*				5	5	P	0.016
			*Butyriboletus*	*B. appendiculatus*			2		2	U	n/a
				*B. pseudospeciosus*	12		3		15	U	0.075
				*B. subappendiculatus*			6	4	10	U, N	0.049
			*Caloboletus*	*C. radicans*			2		2	U	n/a
			*Harrya*	*H. chromapes*				3	3	U, N	0.005
			*Heimioporus*	*H. japonicus*				2	2	P	n/a
			*Neoboletus*	*N. multipunctatus*		4	2	3	9	U	0.045
				*N. obscureumbrinus*				1	1	U	n/a
			*Pulveroboletus*	*P. brunneopunctatus*				1	1	U	n/a
			*Retiboletus*	*R. retipes*				2	2	E	n/a
			*Rugiboletus*	*R. extremiorientalis*	4	1			5	U	0.01
			*Sutorius*	*S. uridiformis*			1	8	9	U	0.078
			*Tylopilus*	*T. microsporus*	4				4	P	0.014
				*T. neofelleus*				1	1	p	n/a
				*T. obscurus*				1	1	U, N	n/a
		*Boletinellaceae*	*Phlebopus*	*P. portentosus*				1	1	E	n/a
		*Gomphidiaceae*	*Chroogomphus*	*C. rutilus*	1	1	2		4	E	0.039
		*Gyroporaceae*	*Gyroporus*	*G. ballouii*	2				2	U	n/a
		*Sclerodermataceae*	*Scleroderma*	*S. yunnanense*	14				14	E	0.002
		*Suillaceae*	*Suillus*	*S. bovinus*	2				2	M	n/a
	*Cantharellales*	*Hydnaceae*	*Cantharellus*	*C. amethysteus*	2	3	1	1	7	E	0
				*C. cibarius*	62	7	19	2	90	E	0
				*C. cinereus*				4	4	E	0
				*C. cinnabarinus*	18	1	3	5	27	E	0.034
				*C. enelensis*		4	1	4	9	U, N	0.011
				*C. formosus*	1	0	1	0	2	E	n/a
				*C. friesii*			2	2	4	U	0.025
				*C. lateritius*	2	0	0	1	3	E	0.009
				*C. lewisii*	4	0	7	2	13	U, N	0.051
				*C. pallens*			2		2	E	0.078
				*C. roseocanus*			2	1	3	U	0.006
				*C. subalbidus*	13	8	0	2	23	E	0.034
				*C. tenuithrix*				1	1	U	n/a
			*Craterellus*	*C. cornucopioides*		0	4	0	4	E	0.011
				*C. luteus*	2	0	0	2	4	E	0.006
	*Gomphales*	*Gomphaceae*	*Gomphus*	*G. clavatus*				5	5	E	0.024
			*Ramaria*	*R. apiculata*				5	5	E	0.0026
				*R. araiospora*				5	5	E	0.061
				*R. aurantiisiccescens*				2	2	U	n/a
				*R. aurea*				1	1	M	n/a
				*R. botrytis*			8	8	16	E	0.064
				*R. conjunctipes*				1	1	E	n/a
				*R. cystidiophora*				3	3	U	0.095
				*R. flavobrunnescens*				1	1	E	n/a
				*R. formosa*				7	7	M	0.041
				*R. fumigata*				5	5	P	0.051
				*R. obtusissima*				13	13	E	0.044
				*R. pinicola*				1	1	U, N	n/a
				*R. rubrievanescens*	1	18	5	2	26	U, N	0.012
	*Hymenochaetales genera incertae sedis*		*Trichaptum*	*T. abietinum*	2				2	U	n/a
	*Polyporales*	*Cerrenaceae*	*Cerrena*	*C. unicolor*			2		2	E	n/a
		*Grifolaceae*	*Grifola*	*G. frondosa*	1	1			2	E	n/a
		*Polyporaceae*	*Amauroderma*	*A. rugosum*	16			3	19	U	0.055
	*Russulales*	*Albatrellaceae*	*Albatrellus*	*A. confluens*				4	4	E	0.028
		*Russulaceae*	*Lactarius*	*L. deliciosus*	7				7	E	0
				*L. deterrimus*	5	1	2		8	E	0.02
				*L. fulvissimus*			1		1	E	n/a
				*L. hatsudake*	10				10	E	0.015
				*L. piperatus*				1	1	M	n/a
				*L. quieticolor*	3				3	E	0.026
				*L. sanguifluus*	3	4	1		8	E	0.029
				*L. semisanguifluus*			1		1	U	n/a
				*L. bertillonii*			1		1	U	n/a
				*L. dwaliensis*			1		1	U, N	n/a
				*L. glaucescens*		1	3		4	U, N	0.034
				*L. leae*			1		1	U	n/a
				*L. piperatus*	2	3	2	1	8	M	0.018
				*L. rugatus*			3	1	4	E	0.005
				*L. subvolemus*			1		1	U, N	n/a
				*L. tenuicystidiatus*				3	3	E	0.002
				*L. volemus*	10	4	12	8	34	E	0.016
			*Russula*	*R. aeruginea*			2		2	E	n/a
				*R. albonigra*				2	2	E	n/a
				*R. amoenolens*				1	1	U	n/a
				*R. aquosa*				1	1	U	n/a
				*R. aurea*			2	1	3	M	0.017
				*R. aurora*				4	4	E	0.006
				*R. cyanoxantha*	1	15	22	5	43	E	0.017
				*R. densifolia*		1			1	E	n/a
				*R. graminea*			3		3	U	0.07
				*R. minutula*				1	1	U	n/a
				*R. nobilis*				2	2	P	n/a
				*R. paludosa*				1	1	E	n/a
				*R. pubescens*			1	2	3	P	0.104
				*R. renidens*				1	1	U	n/a
				*R. rosea*	1	3	1		5	E	0.059
				*R. stenocystidiata*		5	1	1	7	E	0.059
				*R. turci*				1	1	E	n/a
				*R. versicolor*	2				2	U	n/a
				*R. vinosa*			2		2	E	n/a
				*R. vinosobrunnea*		1			1	U	n/a
				*R. virescens*	190	8	3	4	205	E	0
			*Scutiger*	*S. pes-caprae*				9	9	E	0.029
	*Thelephorales*	*Bankeraceae*	*Sarcodon*	*S. leucopus*	8				8	E	0.002
		*Bankeraceae*		*S. squamosus*	2				2	U, N	n/a
		*Thelephoraceae*	*Pseudotomentella*	*P. mucidula*				1	1	U	n/a
			*Thelephora*	*T. aurantiotincta*	1		8		9	E	0.068
				*T. ganbajun*	410		3		413	E	0.032
				*T. vialis*	14	99	1		114	E	0
Total	11	31	56	159	1074	293	492	339	2198		

The edibility status of each taxon is included. E: edible, P: poisonous, U: edibility uncertain, M: medical use (antitumor, antioxidant, anti-inflammation, immunomodulation). N: new records to China.

**Table 3:** Summary of the conservative cutoff values for phylogenetic species identification and species numbers indicated by both ITS blast and phylogenetic analyses in the selected species groups.

	No. Sequences	No. Genotypes	No. Species by ITS Blast	Cutoff Values for the Phylogenetic Species Identification	No. Provisional Species	No. New Phylogenetic Species
*Boletus* and allies	162	N/A	24	0.05	33	2
*Cantharellus cibarius* species complex	95	15	6	0.028	6	1
*Lactarius*	88	N/A	17	0.022	32	5
*Lyophyllum*	96	N/A	3	0.014	20	2
*Russula virescens* and allies	226	19	1	0.01	5	4
*Termitomyces clypeatus* species complex	380	110	7	0.035	10	6
*Thelephora ganbajun* and allies	489	94	1	0.025	4	0

## Data Availability

Representative ITS sequence for all the OTUs have been submitted to GenBank, the detailed information on the accession numbers and the taxonomic differences between GenBank and UNITE for the same barcode sequences was given in supplementary Table S4.

## Supplementary Materials

The following are available online at https://www.mdpi.com/article/10.3390/jof7040310/s1, Figure S1: A Bayesian tree of the ITS sequences of *Boletus* and allies from Yunnan mushroom markets and their closely related ITS sequences from GenBank, Figure S2: A Bayesian tree of the ITS sequences of the *Cantharellus cibarius* species complex from Yunnan mushroom markets and the closely related ITS sequences from GenBank. Only representative sequences of unique ITS sequences (Genotype) are shown; numbers in the brackets are the number of individuals in each genotype, Figure S3: A Bayesian tree of the ITS sequences of the genus *Lactarius* from Yunnan mushroom markets and the closely related ITS sequences from GenBank, Figure S4: A Bayesian tree of the ITS sequences of the genus *Lyophyllum* from Yunnan mushroom markets and the closely related ITS sequences from GenBank, Figure S5: A Bayesian tree of the ITS sequences of *Russula virescens* and related species from Yunnan mushroom markets and the closely related ITS sequences from GenBank. Only representative sequences of unique ITS sequences (Genotype) are shown; numbers in the brackets are the number of individuals in each genotype, Figure S6: A Bayesian tree of the ITS sequences of the *Termitomyces clypeatus* species complex and allies from Yunnan mushroom markets and their closely related ITS sequences from GenBank. Only representative sequences of unique ITS sequences (Genotype) are shown; numbers in the brackets are the number of individuals in each genotype, Figure S7: A Bayesian tree of the ITS sequences of the species *Thelephora ganbajun* and allies from Yunnan and the closely related ITS sequences from GenBank. Only representative sequences of unique ITS sequences (Genotype) are shown; numbers in the brackets are the number of individuals in each genotype, Figure S8: A Bayesian tree of the TEF-1 sequences of the species *Thelephora ganbajun* and allies from Yunnan and the closely related TEF-1 sequences from GenBank. Only representative sequences of unique TEF-1 sequences (Genotype) are shown; numbers in the brackets are the number of individuals in each genotype, Table S1: Geographical distribution of mushroom samples analyzed in this study, Table S2: List of species names, strain labels, and GenBank accession numbers of reference ITS sequences used in the phylogenetic analysis, Table S3: Average evolutionary divergence over ITS sequence pairs within and between groups (provincially adopted phylogenetic species) calculated by MEGA 6 using the K2P model, Table S4: List of specimen name, GenBank accession numbers for ITS sequence for all the representative OTUs and the taxonomic differences between GenBank and UNITE for the same barcode sequences.
