# Supplementary material for: Exploring the Species Diversity of Edible Mushrooms in Yunnan, Southwestern China, by DNA Barcoding
Source: J Fungi (Basel). 2021 Apr 17;7(4):310. doi: 10.3390/jof7040310 (PMC8074183; doi:10.3390/jof7040310)
Supplement: Supplementary file 1 [file jof-07-00310-s001.zip › Supplementary materials/Table S2 List of species names, strain lables and GenBank accession numbers of reference ITS sequences used in the phylogenetic analysis..pdf]

Table S2 List of species names, strain labels and GenBank accession numbers of reference ITS sequences used in the phylogenetic analysis.

Species	Strain Number	GenBank Number
Aureoboletus roxanae	Mushroom Observer #294173	MK547058.1
Aureoboletus roxanae	S.D. Russell MycoMap # 571	MN906209.1
Aureoboletus roxanae	S.D. Russell MycoMap # 7613	MN906210.1
Aureoboletus roxanae	CFMR:BZ-2120 TJB-9401	MN250219.1
Aureoboletus roxanae	CFMR:BZ-2120 TJB-9400	MN250207.1
Boletus brunneissimus	Kun2	DQ407249.1
Boletus edulis	ASIS23225	KF692085.1
Boletus edulis	YNCX04	GQ900594.1
Boletus edulis	YNB200	GQ900593.1
Boletus edulis	Q33	KC414231.1
Boletus edulis	CAR86	KP031594.1
Boletus edulis	LE 287795	KC422613.1
Boletus edulis	GO-2009-283	KC152074.1
Boletus edulis	GO-2009-282	KC152072.1
Boletus edulis	F55586	AB821458.1
Boletus edulis	-	GQ984158.1
Boletus edulis	YM4	EF646277.1
Boletus edulis	Chu11	DQ397949.1
Boletus edulis	F55587	AB821459.1
Boletus edulis	MY4415	AB827926.1
Boletus edulis	YM6	EF646278.1
Boletus edulis	375	EU554664.1
Boletus luridiformis	WA0000052289	KX756398.1
Boletus luridiformis	WA0000052272	KX756397.1
Boletus luridiformis	AAM630/06	FM958177.1
Boletus luridiformis	3N	MK492598.1
Boletus magnificus	Chu19	DQ407260.1
Boletus magnificus	Kun4	DQ407251.1
Boletus recapitulatus	-	KP938967.1
Boletus reticulatus	LE 234822	KC422578.1
Boletus reticulatus	LE 203191	KC422595.1
Boletus reticulatus	LE 217010	KC422584.1
Boletus reticulatus	LE 287798	KC422616.1
Boletus rubripes	OMS 1000895	KT968606.1
Boletus rubripes	JLF2070	KC812294.1
Boletus rubripes	JLF2078	KC812296.1
Boletus rubropunctus	AB08	MH796988.1
Boletus rubropunctus	JLF5666	MH190826.1
Boletus rubropunctus	FLAS-F-60615	MH016805.1
Boletus rubropunctus	FLAS-F-60153	MF074842.1
Boletus rubropunctus	VBHI-F473a	MF161282.1

Boletus rubropunctus	FLAS-F-60324	MF153071.1
Boletus rubropunctus	FLAS-F-60909	MH016884.1
Boletus rubropunctus	FLAS-F-60194	MF074781.1
Boletus shiyong	Groc-11407 clone 2	KT693261.1
Boletus shiyong	Groc-11407 clone 1	KT693260.1
Boletus shiyong	DJJ	KY741856.1
Boletus shiyong	HKAS57472	KM820794.1
Boletus shiyong	HKAS82472	KM820795.1
Boletus sinoedulis	HKAS 53613	NR_158292.1
Boletus vermiculosoides	Mushroom Observer #281329	MH236222.1
Boletus vermiculosoides	S.D. Russell MycoMap 6632	MK560107.1
Butyriboletus huangnianlaii	FHMU 2207	NR_163761.1
Butyriboletus huangnianlaii	N.K.Zeng3245	MH885350.1
Butyriboletus roseoflavus	Arora977	KC184432.1
Butyriboletus roseoflavus	HKAS63609	KJ909518.1
Butyriboletus roseoflavus	HKAS63593	KJ909517.1
Butyriboletus roseoflavus	HKAS54099	KJ909519.1
Butyriboletus yicibus	HKAS57668	KJ909522.1
Butyriboletus yicibus	HKAS 57503	KT002608.1
Butyriboletus yicibus	HKAS56163	KJ909520.1
Butyriboletus yicibus	HKAS68010	KJ909521.1
Cantharellus amethysteus	349/07/284	JN944020.1
Cantharellus cascadensis	OSC 75908	AY041181.1
Cantharellus cibarius	AFTOL-ID 607	*DQ200926.1*
Cantharellus cibarius	DARD-115	KJ184345.1
Cantharellus cibarius	PKSR7	KF636761.1
Cantharellus cibarius	WA0000071071	DQ200926.1
Cantharellus cibarius	351/07/300	JN944019.1
Cantharellus cibarius	DM1022	MT644929.1
Cantharellus cibarius	CANCIB1	MK029339.1
Cantharellus cibarius	CL-67	MK281467.1
Cantharellus cibarius	LL-01	MT566420.1
Cantharellus cibarius	236	HQ270128.1
Cantharellus cinnabarinus	ASIS24870	KF692074.1
Cantharellus cinnabarinus	GRSM 77031	DQ898692.1
Cantharellus cinnabarinus	BB 07.053	KF294559.1
Cantharellus cinnabarinus	341/O7.120	JN944016.1
Cantharellus cinnabarinus	BA 05.12.24	*FR682089.1*
Cantharellus decolorans	BB 08.278	KF981367.1
Cantharellus enelensis	Cassia E3 (UWO) haplotype A	KX592717.1
Cantharellus enelensis	Cassia W3	KX592721.1
Cantharellus enelensis	Cassia E3 (UWO) haplotype B	KX592718.1
Cantharellus enelensis	Cassia E5 (UWO)	KX592719.1
Cantharellus flavus	C068	JX030468.1

Cantharellus flavus	CH1	JX030456.1
Cantharellus flavus	C067	JX030467.1
Cantharellus flavus	CH5	JX030457.1
Cantharellus formosus	Sporocarp6-2018	MK673251.1
Cantharellus formosus	JLY2017.05	MH718256.1
Cantharellus formosus	AB17003	MH718255.1
Cantharellus formosus	SAT-13-298-20	KX592752.1
Cantharellus formosus	OSC 76054	AY041184.1
Cantharellus lateritius	MSR4	HQ270122.1
Cantharellus lateritius	161	HQ270121.1
Cantharellus lateritius	119	HQ270117.1
Cantharellus lewisii	1395/BB	MG450669.1
Cantharellus lewisii	394/BB	MG450668.1
Cantharellus lewisii	314/O7.003	JN944021.1
Cantharellus lewisii	PC 314/07.003	NR_120022.1
Cantharellus pallens	1115/BB 12.082	KX907211.1
Cantharellus pallens	1110/BB 12.077	KX907210.1
Cantharellus pallens	997/BB 09.418	KX907206.1
Cantharellus pallens	998/BB 09.430	KX907207.1
Cantharellus pallens	TTS46	MW386299.1
Cantharellus pallens	DM294A	MT773334.1
Cantharellus roseocanus	SAT-12-299-06	KX592757.1
Cantharellus roseocanus	UBC F23802	KX592760.1
Cantharellus roseocanus	CC29	JX030469.1
Cantharellus roseocanus	JLF3397	*MK256925.1*
Cantharellus roseocanus	GO-2009-270	KC152078.1
Cantharellus roseocanus	JLF6638	MK680154.1
Cantharellus roseocanus	JLF6605	MK680153.1
Cantharellus sebosus	BB 08.162	KF981371.1
Cantharellus sebosus	BB 08.234	KF981370.1
Cantharellus subalbidus	HC-PNNT-252	*KT874977.1*
Cantharellus subalbidus	HC-PNNT-022	KT874978.1
Cantharellus subincarnatus	BB 06.096	KF981372.1
Cantharellus tabernensis	323/O7.040	JN944013.1
Cantharellus tabernensis	325/07.042	JN944014.1
Cantharellus tricolor	68/06.180	JN944015.1
Gyrodon lividus	REG Gl1	DQ534568.1
Gyrodon lividus	17191	JF908786.1
Gyroporus cyanescens	NAMA190	EU819495.1
Gyroporus cyanescens	MB05-04	EU718102.1
Gyroporus cyanescens	MCVE:28580	KT363684.1
Harrya chromapes	MQ18R109-QFB30625	MN992166.1
Harrya chromapes	JLF5633	MH191111.1
Harrya chromapes	ITS199	KC552019.1

Hydnum albomagnum	AS231	MH379943.1
Hydnum albomagnum	NAMA2015-050	MH379908.1
Lactarius acicularis	LTH370	HQ318270.1
Lactarius acicularis	KVP08007	HQ318230.1
Lactarius acicularis	LTH265	HQ318277.1
Lactarius acicularis	KVP08002	HQ318226.1
Lactarius acicularis	KVP08033	HQ318242.1
Lactarius akahatsu	sp141	EF685097.1
Lactarius akahatsu	HA4	JX852630.1
Lactarius akahatsu	KUN:F63055	KT163422.1
Lactarius akahatsu	GENT:KW 013	KJ769671.1
Lactarius akahatsu	LTH162	EF141544.1
Lactarius crocatus	KVP08-035	JN388985.1
Lactarius crocatus	KVP08036	HQ318244.1
Lactarius crocatus	KVP08042	HQ318246.1
Lactarius crocatus	KVP08034	HQ318243.1
Lactarius crocatus	LTH245	HQ318234.1
Lactarius crocatus	LTH266	HQ318265.1
Lactarius deliciosus	wrNZ	KY687508.1
Lactarius deliciosus	NZ-Pp	MT397068.1
Lactarius deliciosus	RM Daehncke 3118	KJ769672.1
Lactarius deliciosus	wr98	KY661914.1
Lactarius gerardii	KIINA126	GU258227.1
Lactarius gerardii	D.P.Lewis6983	GU258272.1
Lactarius gerardii	TMI15558	GU258230.1
Lactarius gerardii	X.H.Wang1768	GU258225.1
Lactarius gerardii	A.Verbeken05-283	GU258259.1
Lactarius glaucescens	H.T.Le 66 (GENT)	GU258298.1
Lactarius glaucescens	F:PRL5812	GQ166898.1
Lactarius glaucescens	AV04202gl	HQ318280.1
Lactarius glaucescens	F:PRL5812	GQ166898.1
Lactarius glaucescens	NEHU.MBSR.07	KM282287.1
Lactarius hatsudake	sp159	EF685098.1
Lactarius hatsudake	K62	EF685076.1
Lactarius hatsudake	BH3	JX852624.1
Lactarius hatsudake	hat38541	EF685063.1
Lactarius hatsudake	JN2011-065	KF432967.1
Lactarius hatsudake	wr122	KY661921.1
Lactarius hatsudake	wr86	KY661918.1
Lactarius hatsudake	KY661917.1	KY661917.1
Lactarius hatsudake	FH 12-052	KR364085.1
Lactarius hatsudake	KUN:F83945	KT163424.1
Lactarius hatsudake	LTH167	EF141545.1
Lactarius hengduanensis	XHW2242	KY174922.1

Lactarius hengduanensis	KY174928.1	KY174928.1
Lactarius hengduanensis	XHW3191	KY174927.1
Lactarius hengduanensis	XHW2822	KY174919.1
Lactarius hengduanensis	WR dc40	KY174926.1
Lactarius hengduanensis	WR lt54	KY174924.1
Lactarius hengduanensis	XHW2816	KY174918.1
Lactarius hengduanensis	WR dc39	KY174925.1
Lactarius hygrophoroides	EIU-ASM10004	JQ358910.1
Lactarius hygrophoroides	clone X3_4	JN129397.1
Lactarius hygrophoroides	AV05251hy	HQ318285.1
Lactarius hygrophoroides	EIU-ASM10421	JQ358914.1
Lactarius kesiyae	KW210	KR025617.1
Lactarius kesiyae	KW219	KR025616.1
Lactarius kesiyae	KW346	KF432993.1
Lactarius kesiyae	AV12-022	KR025618.1
Lactarius kesiyae	KW034	KR025621.1
Lactarius longipilus	LTH273	HQ318276.1
Lactarius longipilus	LTH184	HQ318256.1
Lactarius longipilus	LTH168	HQ318235.1
Lactarius longipilus	LTH206	HQ318258.1
Lactarius pinguis	LTH255	HQ318263.1
Lactarius pinguis	LTH117	HQ318211.1
Lactarius pinguis	SFC20120907-20	MG551741.1
Lactarius pinguis	WA0000072180	MT252532.1
Lactarius piperatus	619	JF908270.1
Lactarius piperatus	M. Lecomte:2001 10 02 15	KF220042.1
Lactarius piperatus	GENT:A. Verbeken 93-023 P1	KF220084.1
Lactarius piperatus	GENT:A. Fraiture 2584	KF220080.1
Lactarius piperatus	M. Lecomte:2000 08 28 41	KF220091.1
Lactarius piperatus	M. Lecomte:2007 06 28 01	KF220085.1
Lactarius piperatus	M. Lecomte:2007 06 28 01	KF220092.1
Lactarius piperatus	M. Lecomte:2003 06 29 01	KF220089.1
Lactarius piperatus	GENT:78111	KF220122.1
Lactarius piperatus	GENT:R. Walleyn 3064	KF220079.1
Lactarius piperatus	LTH51pip	HQ318281.1
Lactarius piperatus	LTH51pip	HQ318281.1
Lactarius pseudohatsudake	ZJP36	KY174908.1
Lactarius pseudohatsudake	HYJ12	KY174903.1
Lactarius pseudohatsudake	ZQ855	KY174905.1
Lactarius pseudohatsudake	ZQ688	KY174910.1
Lactarius pseudohatsudake	XHW2809	KY174904.1
Lactarius pseudohatsudake	XHW3214	KY174907.1
Lactarius pseudohatsudake	QJ10	KY174900.1
Lactarius pseudohatsudake	ZQ665	KY174902.1

Lactarius pseudohatsudake	ZXT14	KY174901.1
Lactarius pseudohatsudake	QJ23	KY174916.1
Lactarius small	-	KY684136.1
Lactarius small	-	KY684135.1
Lactarius small	-	KY684134.1
Lactarius volemus	LTH264	HQ318264.1
Lactarius volemus	LTH219	HQ318254.1
Lactarius volemus	KVP08032	HQ318241.1
Lactarius volemus	WA0000072261	MT252589.1
Lactarius volemus	KVP08039	HQ318245.1
Lactarius volemus	KVP08026	HQ318238.1
Lactarius volemus	LTH247	HQ318261.1
Lactarius volemus	IB-2019a	HQ318233.1
Lanmaoa pallidorosea	S.D. Russell Mushroom	MN906175.1
Lanmaoa pallidorosea	S.D. Russell MycoMap # 6778	MN906177.1
Lanmaoa pallidorosea	S.D. Russell Mushroom	MN906174.1
Leccinum rugosiceps	Kun2	DQ407250.1
Leccinum rugosiceps	A8	MG383655.1
Leccinum rugosiceps	YNLR-1	KY440111.1
Leccinum rugosiceps	MHHNU 8338	KU518328.1
Lentinus squarrosulus	AO-DEBCR-2	KT207468.1
Lentinus squarrosulus	WCR1201	KT956127.1
Lentinus squarrosulus	JZ26	MG719283.1
Lentinus squarrosulus	AO-DEBCR-3	KT207470.1
Lepista saeva	AH39154	KJ681015.1
Lepista subaequalis	CBS 373.63	MH858311.1
Lyophyllum connatum	Lcxin	HM119488.1
Lyophyllum connatum	MQ17002-QFB29510-HRL2437	MN992619.1
Lyophyllum connatum	CBS 148.50	MH856577.1
Lyophyllum connatum	2386-QFB-25622	KM406963.1
Lyophyllum connatum	SR-32	HE819396.1
Lyophyllum connatum	FR2013242	KP192563.1
Lyophyllum connatum	MQ18R037-QFB30120	MN992226.1
Lyophyllum connatum	1037	MH930131.1
Lyophyllum decastes	Ldec3Q106	JN983983.1
Lyophyllum decastes	Ldec4Q106	JN983978.1
Lyophyllum decastes	Ldec5Q106	JN983982.1
Lyophyllum decastes	Ldec2Q106	JN983980.1
Lyophyllum decastes	Sundberg091007b	HM572549.1
Lyophyllum decastes	Ldec7Q106	JN983981.1
Lyophyllum favrei	BSI94cp2	AF357035.2
Lyophyllum favrei	-	EF421102.1
Lyophyllum favrei	6334	JF908333.1
Lyophyllum fumosum	-	MK209602.1

Lyophyllum fumosum	Lipovac090903	HM572538.1
Lyophyllum fumosum	CA20081030.09	JX280412.1
Lyophyllum fumosum	L2010512371	JX966310.1
Lyophyllum fumosum	L2010512370	JX966309.1
Lyophyllum fumosum	Sundberg090813	HM572537.1
Lyophyllum fumosum	LAS00-144	HM572541.1
Lyophyllum fumosum	SJ02-006	HM572539.1
Lyophyllum fumosum	CB08330	KT875064.1
Lyophyllum fumosum	Aase810721	HM572540.1
Lyophyllum fumosum	AR09641	KT875066.1
Lyophyllum infumatu	FR2014003	KP192584.1
Lyophyllum infumatu	FR2014070	KP192627.1
Lyophyllum infumatu	FR2013200	KP192537.1
Lyophyllum infumatu	FR2014026	KP192575.1
Lyophyllum infumatu	284487	MN173357.1
Lyophyllum infumatu	FR2013256	KP192568.1
Lyophyllum leucopha	FR2014021	KP192606.1
Lyophyllum leucopha	HMAS 290452	MK966520.1
Lyophyllum leucopha	FR2014002	KP192581.1
Lyophyllum leucopha	HMAS 290357	MK966521.1
Lyophyllum moncalvo	96328	KJ461904.1
Lyophyllum moncalvo	102581	KJ461912.1
Lyophyllum moncalvo	72796	KJ461890.1
Lyophyllum moncalvo	96332	KJ461906.1
Lyophyllum shimeji	haukebo1982	HM572529.1
Lyophyllum shimeji	Olsen821006	HM572530.1
Lyophyllum shimeji	SAT-10-238-15	MT955155.1
Lyophyllum shimeji	Olausson090922	HM572533.1
Lyophyllum shimeji	Sundberg090813b	HM572532.1
Lyophyllum shimeji	Sundberg091007	HM572528.1
Lyophyllum shimeji	Sundberg090813c	HM572535.1
Lyophyllum shimeji	Sundberg090813c	HM572525.1
Lyophyllum shimeji	Lipovac090911	HM572534.1
Lyophyllum shimeji	Antilla090921	HM572526.1
Lyophyllum shimeji	Eilertsen090908	HM572531.1
Lyophyllum shimeji	Fallman090927	HM572536.1
Lyophyllum shimeji	Karlsson090915	HM572527.1
Lyophyllum shimeji	L2010512377	JX966311.1
Neoboletus multipunctatus	N.K.Zeng3324	MK061359.1
Neoboletus multipunctatus	FHMU 1620	NR_163762.1
Paxillus adelphus	See09.1	KU163507.1
Paxillus adelphus	ALB09.10	KU163468.1
Paxillus adelphus	Tec08.1	KU163510.1
Paxillus rubicundulus	-	NR_147640.1

Paxillus rubicundulus	Orton2905	KU163502.1
Paxillus rubicundulus	LBH08.m1	KU163493.1
Phlebopus portentosus	H-01-3-1	GQ253574.1
Phlebopus portentosus	FG-RZS5	MN962564.1
Phlebopus portentosus	FG-YX2	MT272131.1
Phlebopus portentosus	FG-YX4	MN962546.1
Pulveroboletus flaviscabrosus	-	NR_154348.1
Pulveroboletus flaviscabrosus	HKAS83190	KX453802.1
Pulveroboletus flaviscabrosus	HKAS82616	KX453801.1
Pulveroboletus macrosporus	G.L.Zhang051	KX453813.1
Pulveroboletus macrosporus	HKAS58867	KX453815.1
Pulveroboletus reticulopileus	HKAS 80851	KX453809.1
Pulveroboletus reticulopileus	S.D.Yang112	KX453808.1
Retiboletus fuscus	FHMU1403	MH367481.1
Retiboletus fuscus	FHMU1386	MH367480.1
Retiboletus fuscus	FHMU1454	MH367482.1
Rubroboletus haematinus	JLF2905	KT122394.1
Rubroboletus haematinus	OSC 114190	KT122391.1
Rubroboletus haematinus	DN1714	KT122393.1
Rubroboletus haematinus	JLF1343	KX610678.1
Russula aeruginea	2010BT144	MT738283.1
Russula aeruginea	LAH35404	MT738282.1
Russula aeruginea	ANT152-QFB28552	MN992507.1
Russula andaluciana	39239 (AH)	MK105629.1
Russula andaluciana	BM360	MK105627.1
Russula andaluciana	39198	MK105628.1
Russula atroglauca	SAV F-2166	MT738275.1
Russula atroglauca	SAV F-3066	MT738274.1
Russula atroglauca	JK RUS 11092701	MT738273.1
Russula atroglauca	SAV F-20375	MT738272.1
Russula cf.virescen	NXS61	MT333229.1
Russula cf.virescen	SZ6_19	KC598395.1
Russula cf.virescen	HMJAU 38017	KY681441.1
Russula cf.virescen	NH5_15	KC598474.1
Russula cf.virescen	YP7_12	KC598360.1
Russula cf.virescen	SM1_3	KC598387.1
Russula cf.virescen	HBAU15016	MT337532.1
Russula cf.virescen	NXS81	MT333230.1
Russula cf.virescen	HQR128	MT333231.1
Russula cf.virescen	HBAU15019	MT337531.1
Russula cf.virescen	ASIS21606	KF668302.1
Russula cf.virescen	RMUKK30	KX267660.1
Russula cf.virescen	HBAU15010	MT337530.1
Russula cf.virescen	HBAU15008	MT337528.1

Russula cf.virescen	HBAU15013	MT337529.1
Russula cyanoxantha	FH 12-201	KR364093.1
Russula delica	UBC:F30263	KX812842.1
Russula delica	FH12-272	KF432955.1
Russula heterophyll	209RUF24	AY061681.1
Russula heterophyll	OTU1090	MT594671.1
Russula ionochlora	DNA909	HM189875.1
Russula ionochlora	LUGO:ECC17061003	MW376708.1
Russula ionochlora	DNA871	HM189874.1
Russula ionochlora	FH 2010 BT141	MT738289.1
Russula ionochlora	FH 2009 BT01	MT738288.1
Russula ionochlora	040904-02TT	HQ604836.1
Russula mariae	PC BB2004-213	EU598199.1
Russula mariae	SAV_F-4484	MT017557.1
Russula mariae	SAV_F-4564	MT017559.1
Russula mariae	SAV_F-4493	MT017558.1
Russula mariae	JMP0063	EU819426.1
Russula parazurea	KR-M-0044754	MT005917.1
Russula parazurea	12	MN663162.1
Russula parazurea	MF01.10.2003	DQ422007.1
Russula parvoviresc	AG_15_756	MG934209.1
Russula parvoviresc	AG_16_1223	AY061727.1
Russula parvoviresc	-	*MF193345.1*
Russula prasina	HMAS 279805	MH454352.1
Russula prasina	HMAS 279806	MH454353.1
Russula prasina	HMAS 281232	MH454351.1
Russula substriata	XHW4766	MH724921.1
Russula substriata	XHW4767	MH724922.1
Russula substriata	WJ292	MH724919.1
Russula substriata	XHW4785	MH724923.1
Russula substriata	XHW4749	MH724920.1
Russula vesca	M8	MT469923.1
Russula vesca	654	JF908645.1
Russula vesca	31	JF908637.1
Russula vesca	210RUS24	AY061723.1
Russula violeipes	208IS76	AY061726.1
Russula violeipes	1792	JF908655.1
Russula viridicinna	HBAU15018	MT337525.1
Russula viridicinna	HBAU15014	MT337524.1
Russula viridicinna	FQM128	MT333232.1
Russula viridicinna	HBAU15009	MT337523.1
Russula viridicinna	HFJAU0624	MN258683.1
Russula viridirubrolimbata	HBAU15011	MT337526.1
Russula viridirubrolimbata	HBAU15020	MT337527.1

Russula xanthoviren	B17091630	MG786055.1
Russula xanthoviren	H15060611	MG786056.1
Stereum hirsutum	CBS 930.70	MH860009.1
Stereum hirsutum	SKU512	HM004553.1
Suillellus subvelutipes	RAS451	MT196984.1
Suillellus subvelutipes	CNV13	MT345190.1
Suillellus subvelutipes	NS082512	KX610675.1
Suillellus subvelutipes	Observer #285181	MH244205.1
Suillus pictus	SFC20120922-06	KJ415103.1
Suillus pictus	SFC20120922-06	KU059624.1
Termitomyces bulborhizus	-	HM230663.1
Termitomyces clypeatus	MU19-50	FJ147329.1
Termitomyces clypeatus	MU5-50ssiM16-type2	FJ147330.1
Termitomyces clypeatus	strain MU5-50ssiM31-type1	FJ147331.1
Termitomyces clypeatus	MU5-50ssiM32-type2	FJ147332.1
Termitomyces clypeatus	MU5-50ssiM33-type1	FJ147333.1
Termitomyces clypeatus	MU26-49	GU967667.1
Termitomyces clypeatus	MU27-49	GU967668.1
Termitomyces clypeatus	MU3-51	GU967669.1
Termitomyces clypeatus	MU4-51	GU967670.1
Termitomyces clypeatus	MU7-51	GU967671.1
Termitomyces eurhizus	-	AF321366.1
Termitomyces eurrhizus	isolate D13	KC414235.1
Termitomyces eurrhizus	isolate J5	KC414254.1
Termitomyces heimii	strain JMleg.MUIDs.n	AF357022.2
Termitomyces heimii	PUN4243	JQ928938.1
Termitomyces heimii	-	HM230662.1
Termitomyces radicatus	-	HM230660.1
Termitomyces striatus	-	AF321367.1
Thelephora anthocephala	RA711-8	*MK234216.1*
Thelephora anthocephala	UBC F28410	KP454019.1
Thelephora anthocephala	NSK 1014540	MT773612.1
Thelephora anthocephala	src614	DQ974771.1
Thelephora caryophyllea	IB60087	EF655705.1
Thelephora caryophyllea	ELarsson89-09	MK602776.1
Thelephora caryophyllea	MQ17162-QFB29670	MN992647.1
Thelephora caryophyllea	MQ18R032-QFB30115	MN992284.1
Thelephora caryophyllea	RA711-8	MN992284.1
Thelephora ganbajun	JN1-6	KY245122.1
Thelephora ganbajun	JN1-13	KY245113.1
Thelephora ganbajun	YL3-13	KY245258.1
Thelephora ganbajun	S108	KY245180.1
Thelephora ganbajun	YL3-4	KY245264.1
Thelephora ganbajun	YL3-1	KY245254.1

Thelephora ganbajun	JN1-2	KY245119.1
Thelephora ganbajun	JN1-14	KY245114.1
Thelephora ganbajun	Gb001	EU696791.1
Thelephora ganbajun	JN1-9	KY245124.1
Thelephora ganbajun	JN1-7	KY245123.1
Thelephora ganbajun	GJ1-7	KY245107.1
Thelephora ganbajun	JN1-16	KY245116.1
Thelephora ganbajun	Gb259	EU696927.1
Thelephora palmata	UBC:F33078	MF908479.1
Thelephora palmata	JLF3733	MK847520.1
Thelephora palmata	HA27	KR019858.1
Thelephora palmata	JMP0085	EU819443.1
Thelephora pseudoversatilis	UNAM:FCME26138	KJ462503.1
Thelephora pseudoversatilis	11H2-2	KU530338.1
Thelephora pseudoversatilis	UNAM:FCME26152	KJ462486.1
Thelephora pseudoversatilis	FCME 26232	JX075890.1
Thelephora terrestris	ecmTe1	JX679367.1
Thelephora terrestris	D. 1-IBL	FJ532478.1
Thelephora terrestris	OTU: KGP22	DQ822828.1
Thelephora terrestris	CBS 703.85	MH861911.1
Thelephora versatilis	UNAM:FCME26141	KJ462504.1
Thelephora versatilis	UNAM:FCME26247	KJ462502.1
Thelephora versatilis	UNAM:FCME26144	KJ462501.1
Thelephora versatilis	MEXU:26359	KJ462500.1
Thelephora vialis	Fire Survey 569	MN121029.1
Thelephora vialis	NAMA 2015-276	MN121022.1
Thelephora vialis	Fire Survey 147	MH910560.1
Tylopilus balloui	CMU51-SL-39	KX017306.1
Tylopilus balloui	CMU51-SL-37	KX017305.1
Tylopilus balloui	CMU51-SL-42	KX017307.1
Tylopilus balloui	CMU51-SL-32	KX017304.1
Tylopilus neofelleus	DC 16-64	MG777524.1
Tylopilus neofelleus	YT20090720	KM975489.1
Tylopilus neofelleus	MHHNU 8354	MK253785.1
Tylopilus virens	Kun16	DQ407256.1
Tylopilus virens	-	KX017308.1
Tylopilus virens	HE2757	KC505585.1
Tylopilusneofelleus	DC 16-63	MG777523.1
Xerocomellus redeuilhii	STU:20121007GS10074	KX905051.1
Xerocomellus redeuilhii	1999/0901	KU721024.1
Xerocomellus redeuilhii	1999/1057	KU721023.1
Xerocomellus redeuilhii	FBozok00136	MH472623.1
Xerocomellus redeuilhii	ML411142XE	MH011842.1
Xerocomellus redeuilhii	ML610102XR	MH011929.1

Xerocomus ferrugineus	PNGS/306	KX882676.1
Xerocomus ferrugineus	WU 39588	MT644941.1
Xerocomus ferrugineus	SAT-10-238-19	MT955158.1
Xerocomus ferrugineus	SAT-16-237-02	MT955157.1
Xerocomus ferrugineus	MQ18R259-QFB30775	MN992288.1
