# Supplementary figures and images for: Exploring the Species Diversity of Edible Mushrooms in Yunnan, Southwestern China, by DNA Barcoding
Source: J Fungi (Basel). 2021 Apr 17;7(4):310. doi: 10.3390/jof7040310 (PMC8074183; doi:10.3390/jof7040310)

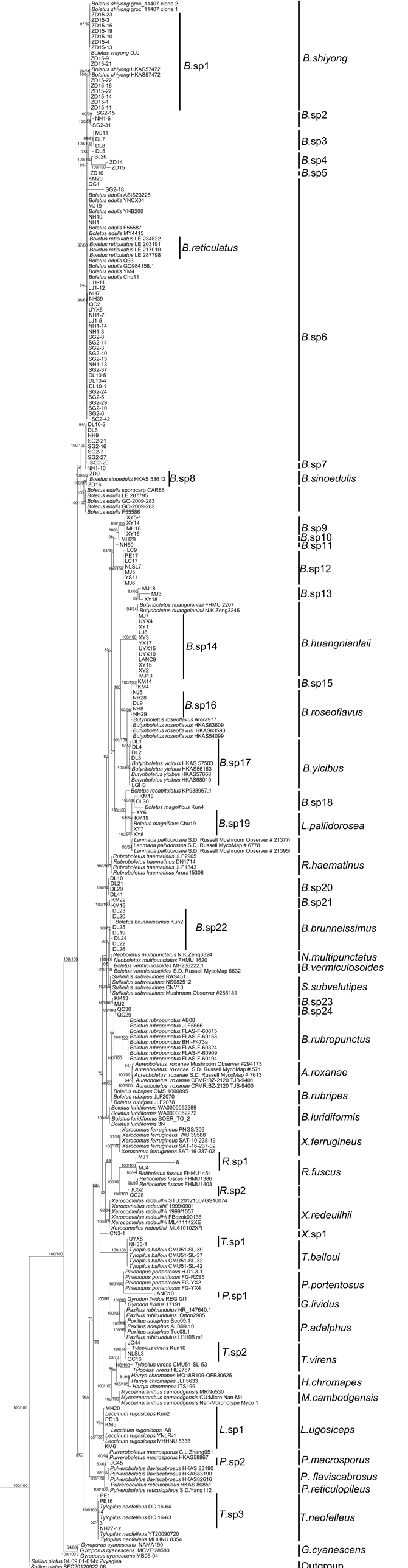

Supplement: Supplementary file 1 [file jof-07-00310-s001.zip › Supplementary materials/Figure S1 Boletus.pdf]

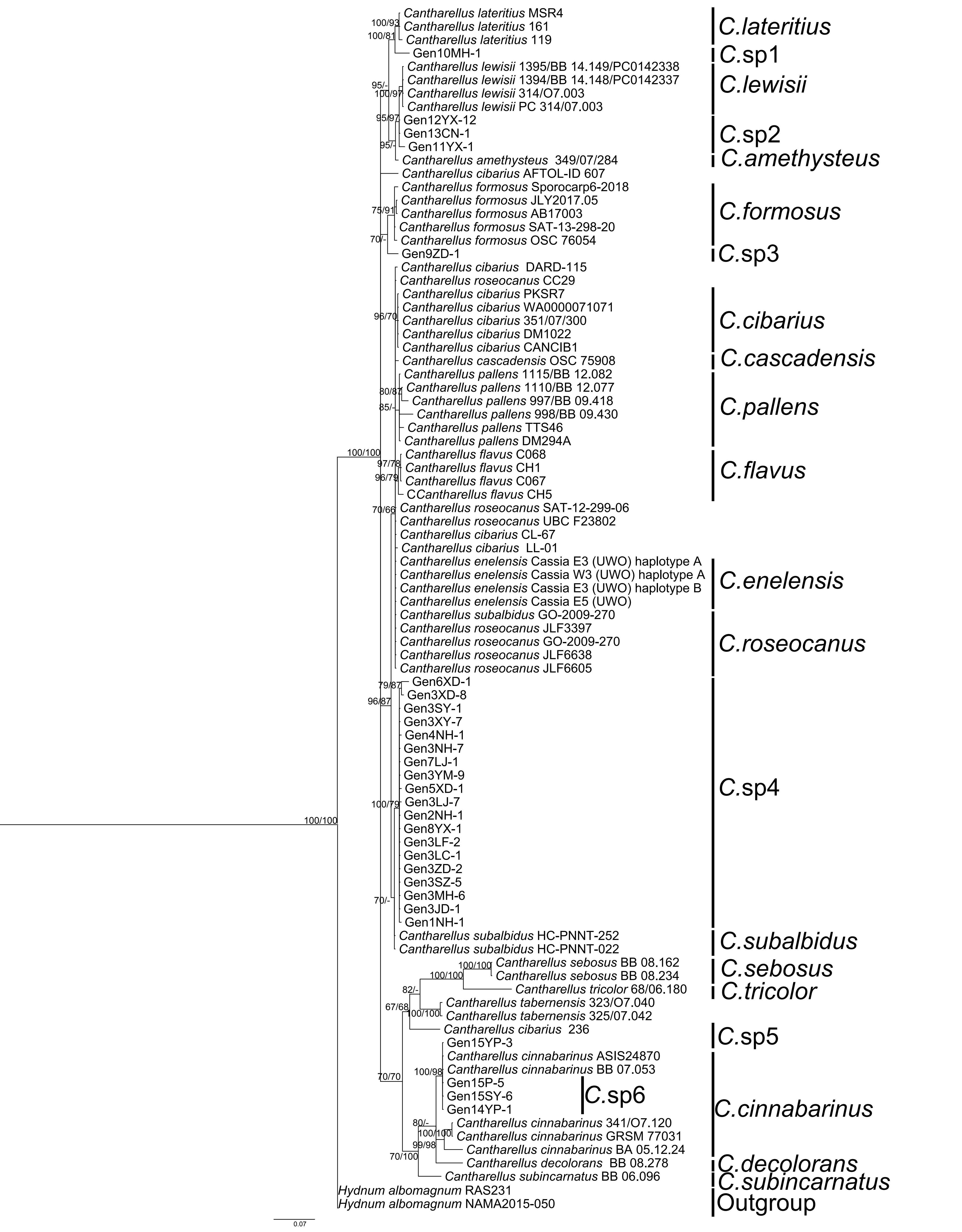

Supplement: Supplementary file 1 [file jof-07-00310-s001.zip › Supplementary materials/Figure S2 Cantharellus.pdf]

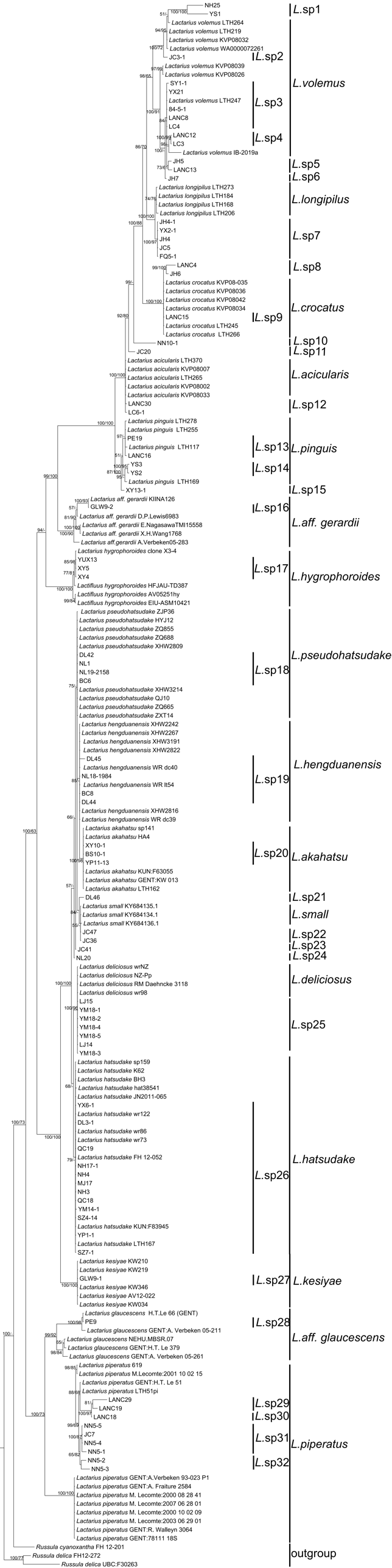

Supplement: Supplementary file 1 [file jof-07-00310-s001.zip › Supplementary materials/Figure S3 Lactarius.pdf]

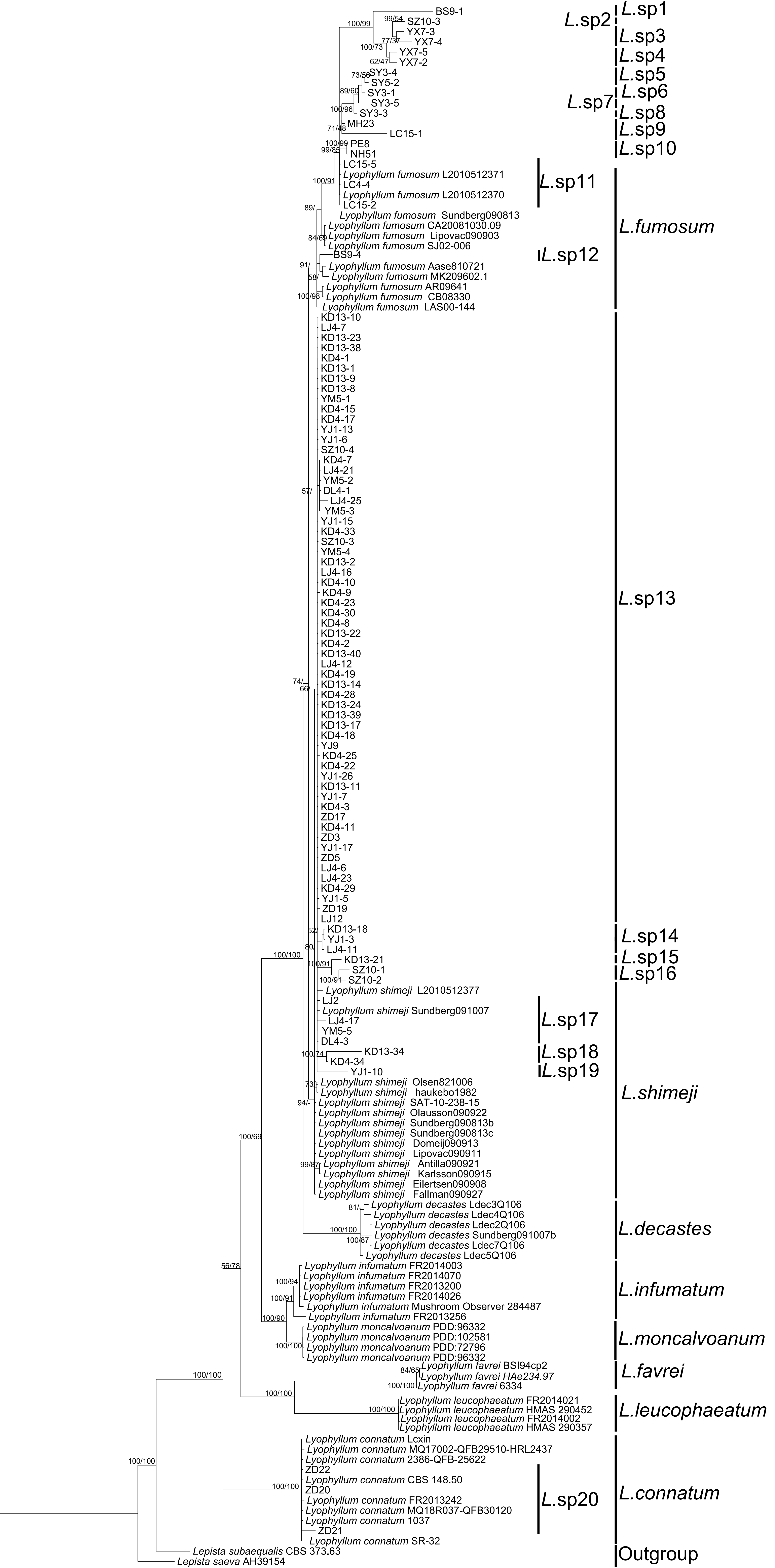

Supplement: Supplementary file 1 [file jof-07-00310-s001.zip › Supplementary materials/Figure S4 Lyophyllum.pdf]

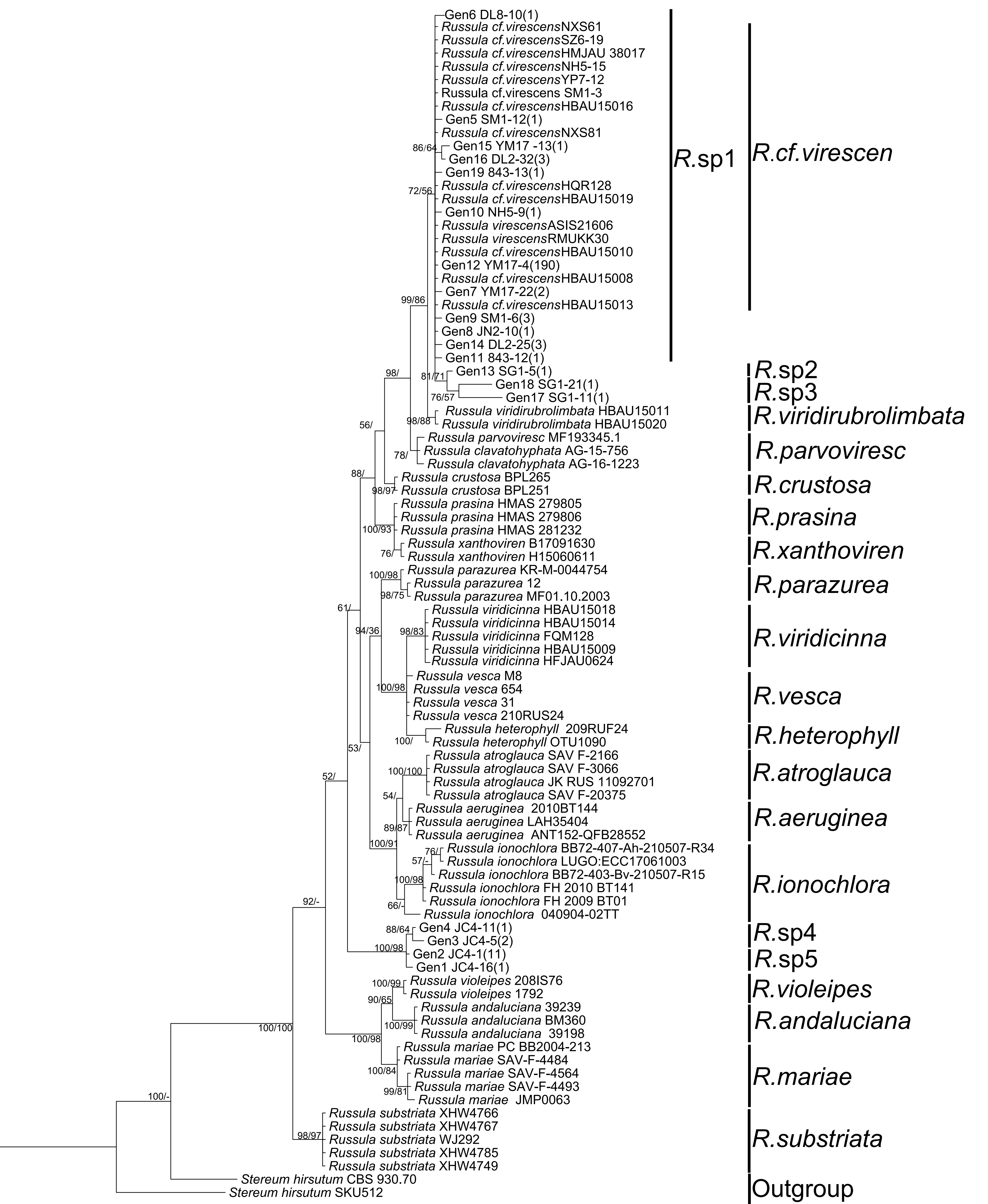

Supplement: Supplementary file 1 [file jof-07-00310-s001.zip › Supplementary materials/Figure S5 Russula.pdf]

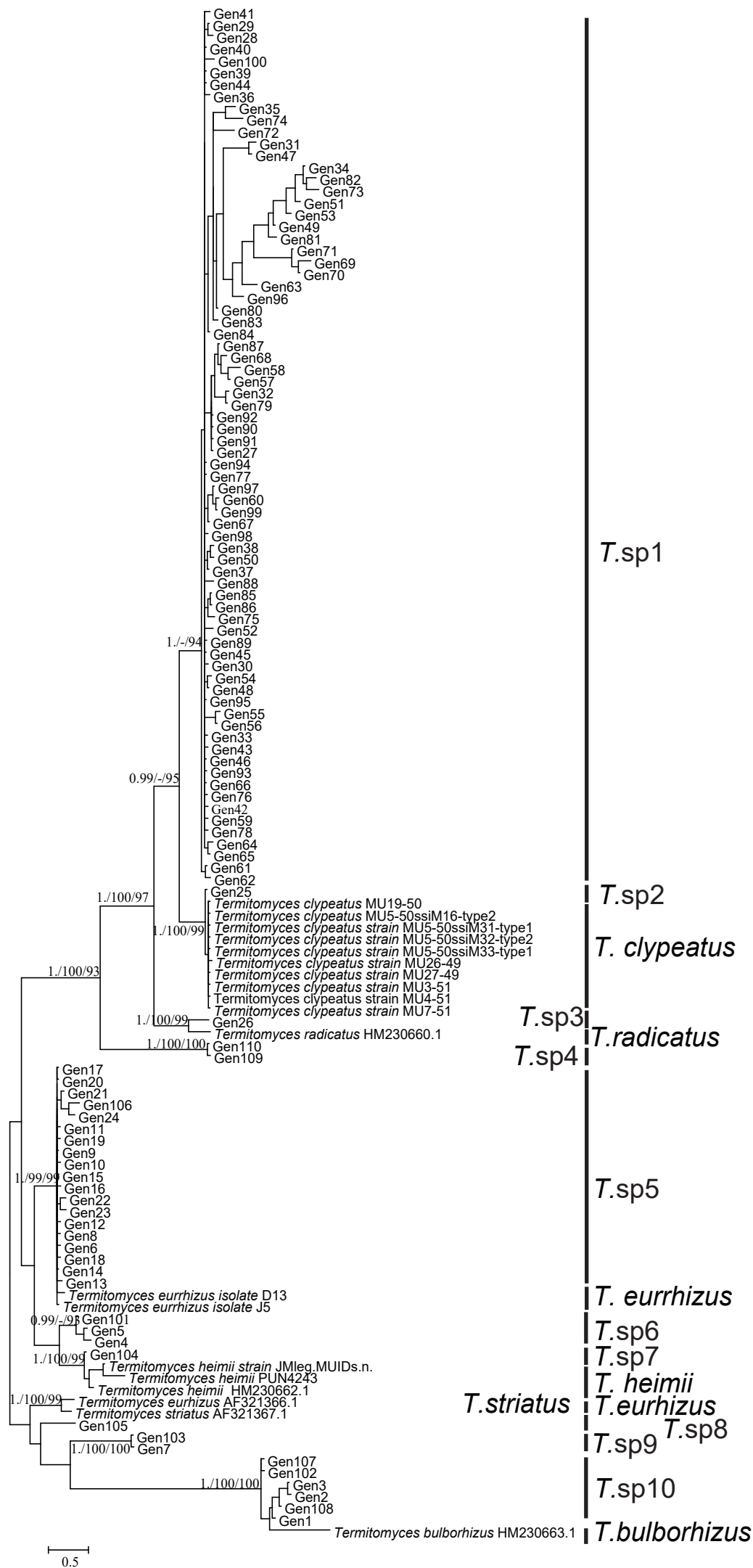

Supplement: Supplementary file 1 [file jof-07-00310-s001.zip › Supplementary materials/Figure S6 Termitomyces.pdf]

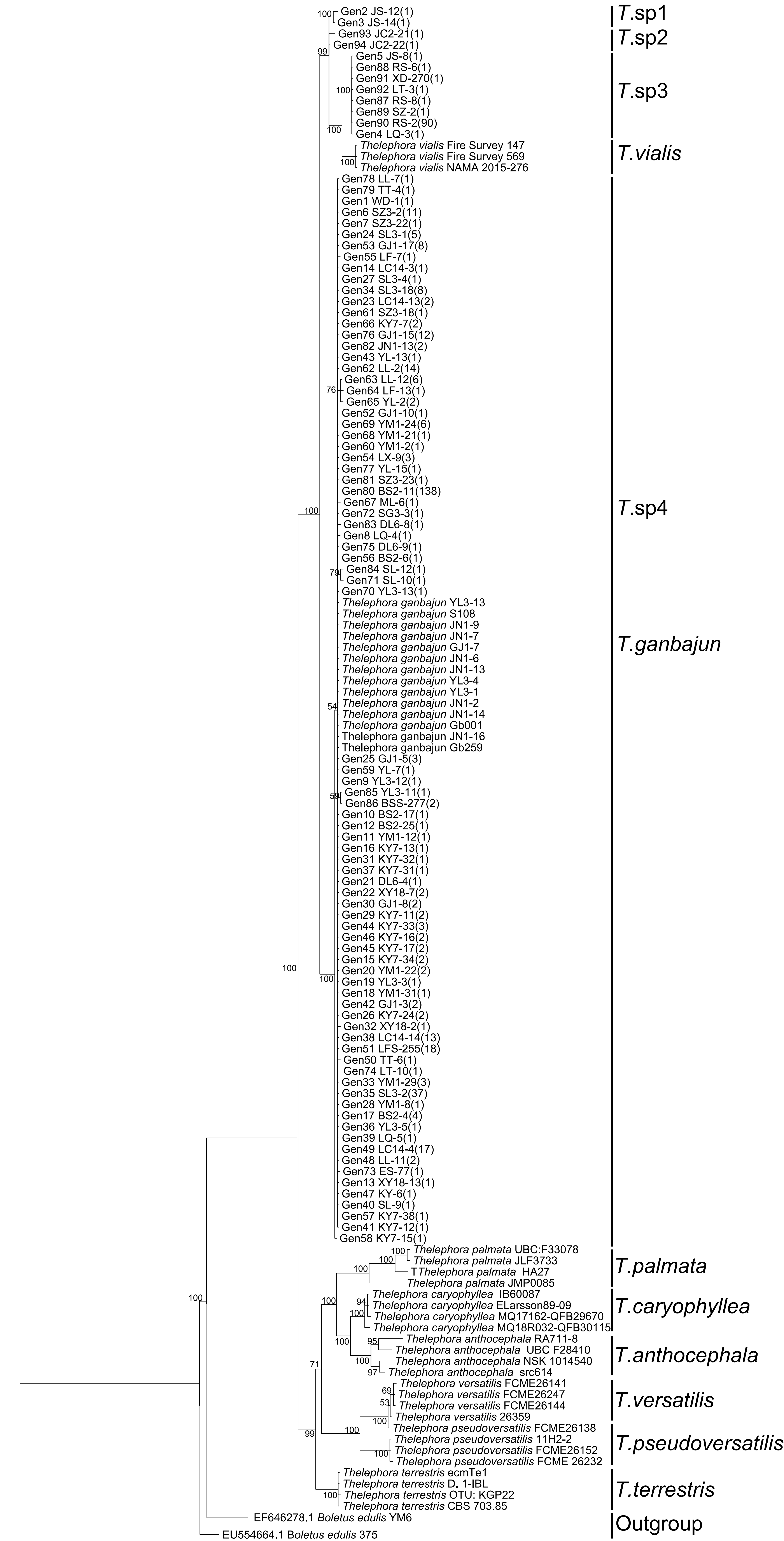

Supplement: Supplementary file 1 [file jof-07-00310-s001.zip › Supplementary materials/Figure S7 Thelephora.pdf]

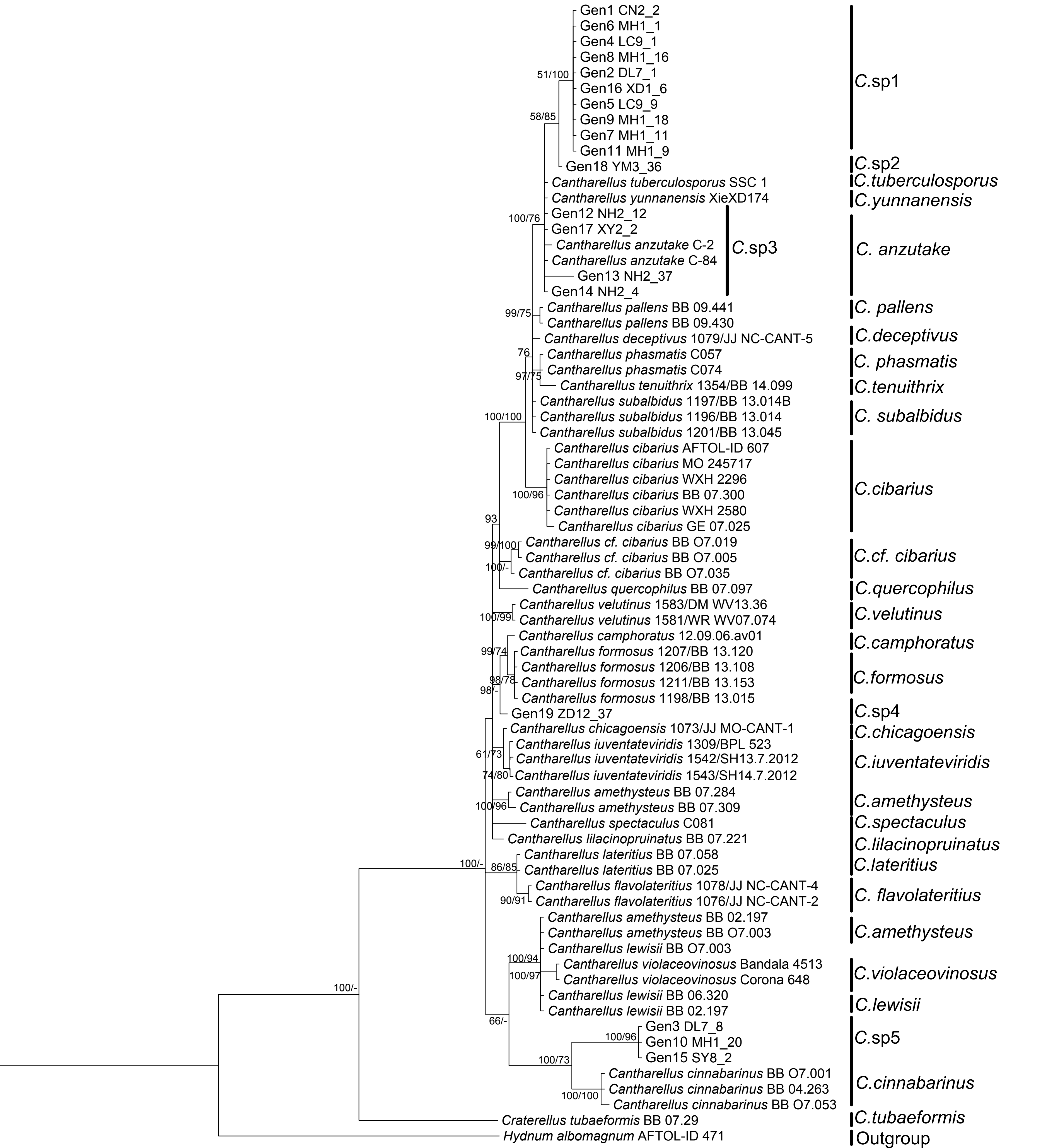

Supplement: Supplementary file 1 [file jof-07-00310-s001.zip › Supplementary materials/Figure S8 Cantharellus_TEF phylogeny.pdf]
